# Systematic Review of COVID-19-Related Physical Activity-Based Rehabilitations: Benefits to Be Confirmed by More Robust Methodological Approaches

**DOI:** 10.3390/ijerph19159025

**Published:** 2022-07-25

**Authors:** Mélina Bailly, Léna Pélissier, Emmanuel Coudeyre, Bertrand Evrard, Rea Bingula, Corinne Rochette, Laurent Mériade, Christelle Blavignac, Anne-Cécile Fournier, Yves-Jean Bignon, Fabrice Rannou, Frédéric Dutheil, David Thivel, Martine Duclos

**Affiliations:** 1 Centre de Recherche en Nutrition Humaine (CRNH), Laboratoire des Adaptations Métaboliques à l’Exercice en Conditions Physiologiques et Pathologiques (AME2P), Unité de Formation et de Recherche (UFR) des Sciences et Techniques des Activités Physiques et Sportives (STAPS), Université Clermont Auvergne, 63000 Clermont-Ferrand, France; melina.bailly@uca.fr (M.B.); david.thivel@uca.fr (D.T.); 2 Service de Médecine Physique et de Réadaptation, Institut National de Recherche pour l’Agriculture, l’Alimentation et l’Environnement (INRAE), Unité de Nutrition Humaine (UNH), Centre Hospitalier Universitaire (CHU) Clermont-Ferrand, Université Clermont Auvergne, 63000 Clermont-Ferrand, France; ecoudeyre@chu-clermontferrand.fr; 3 Service d’Immunologie, Centre Hospitalier Universitaire (CHU) Gabriel-Montpied, 63000 Clermont-Ferrand, France; bevrard@chu-clermontferrand.fr; 4 Micro-Environnement CellulaiRE, Immunomodulation et Nutrition (ECREIN), Institut National de Recherche pour l’Agriculture, l’Alimentation et l’Environnement (INRAE), Unité Mixte de Recherche (UMR) 1019 Unité de Nutrition Humaine (UNH), Université Clermont Auvergne, 63000 Clermont-Ferrand, France; rea.bingula@uca.fr; 5 Health and Territory Chair, Université Clermont Auvergne, CleRMa, 63000 Clermont-Ferrand, France; corinne.rochette@uca.fr (C.R.); laurent.meriade@uca.fr (L.M.); 6 Centre Imagerie Cellulaire Santé, Université Clermont Auvergne, 63000 Clermont-Ferrand, France; christelle.blavignac@uca.fr; 7 Cluster Auvergne-Rhône-Alpes Innovation Innovatherm, 63000 Clermont-Ferrand, France; ac.fournier@innovatherm.fr; 8 U1240 Imagerie Moléculaire et Stratégies Théranostiques, Institut National de la Santé et de la Recherche Médicale (INSERM), Université Clermont Auvergne, 63000 Clermont Ferrand, France; yves-jean.bignon@clermont.unicancer.fr; 9 Département d’Oncogénétique, Centre Jean Perrin, 63011 Clermont-Ferrand, France; 10 Service de Médecine du Sport et des Explorations Fonctionnelles, Centre Hospitalier Universitaire (CHU) de Clermont-Ferrand, Institut National de Recherche pour l’Agriculture, l’Alimentation et l’Environnement (INRAE), Unité de Nutrition Humaine (UNH), Centre de Recherche en Nutrition Humaine (CRNH) Auvergne, Université Clermont Auvergne, 63000 Clermont-Ferrand, France; frannou@chu-clermontferrand.fr (F.R.); mduclos@chu-clermontferrand.fr (M.D.); 11 Preventive and Occupational Medicine, LaPSCo, Physiological and Psychosocial Stress, Centre National de la Recherche Scientifique (CNRS), Centre Hospitalier Universitaire (CHU) Clermont-Ferrand, Witty Fit, Université Clermont Auvergne, 63000 Clermont-Ferrand, France; fdutheil@chu-clermontferrand.fr

**Keywords:** COVID-19, SARS-CoV-2, physical activity, rehabilitation, functional capacity

## Abstract

The first emergency was to receive and treat COVID-19 patients in their acute phase; today, there is a clear need to propose appropriate post-acute rehabilitation programs. The aim of this research was to systematically review the effects of physical activity programs in the recovery of post-COVID-19 patients. The literature search followed the Preferred Reporting Items for Systematic Review and Meta-Analysis (PRISMA) guidelines, was registered in the PROSPERO database (CRD42022289219), and was conducted between August and December 2021. A total of 35 studies out of the 1528 initially identified were finally included in the analysis. The systematic review clearly showed the health benefits of rehabilitation including physical activity in post-COVID-19 recovery, regardless of exercise modalities. These positive results were even observed using minor muscle re-mobilization for severe cases (i.e., postural changes, few steps—2 times/day) or using low volumes of exercise for mild-to-moderate cases (i.e., 120 min/week). A total of 97% of the 29 studies that performed statistical analyses demonstrated a significant increase in at least one parameter of functional capacity, and 96% of the 26 studies that statistically investigated the effects on the quality of life, mental health, and general state reported improvements. Yet, most of the studies were retrospective, uncontrolled, and enrolled aged people with comorbidities presented in severe forms of COVID-19. Physical activity programs, in addition to their high heterogeneity, remained poorly described in 83% of the studies and were part of a multidisciplinary program for 89% of the studies. Despite promising results, there is today a real need for prospective well-designed studies specifically assessing the effects of physical activity. In addition, it might appear relevant to propose standardized programs further considering the main characteristics of patients such as age, comorbidities, or the severity of COVID-19.

## 1. Introduction

In February 2022, the World Health Organization reported more than 430 million confirmed cumulative cases of COVID-19 including more than 5.9 million deaths [1], with approximately 14% of the cases representing severe forms and 5% critical forms [2]. Beyond the acute stage of the Severe Acute Respiratory Syndrome Corona Virus 2 (SARS-CoV-2) infection, many clinical manifestations remain for a high proportion of patients whose recovery is difficult, and even more difficult in the presence of other comorbidities [3]. After hospitalization, more than half of the patients still present symptoms such as fatigue, dyspnea, loss of memory and concentration, or even sleep disorders at least several months after the onset of SARS-CoV-2 [4], highlighting the need for a long-term follow-up of these patients. While the serious consequences of prolonged bed rest/hospitalization and intensive care (post-intensive care syndrome) are well-documented [5,6], it appears that even mild cases of COVID-19 may be associated with persistent symptoms and difficulties in recovery [7]. Indeed, long-COVID-19, called “post-COVID syndrome”, also concerns relatively mild COVID-19 cases [8] and is determined by the time lag between the microbiological recovery (a negative PCR test) and clinical recovery (persistence of various symptoms for weeks, months, or even years) [9]. While focusing on and treating the acute stage was a priority for caregivers and researchers, an increasing number of publications are now discussing the crucial role of appropriate rehabilitation programs (RP), underlying the urgency to provide early, individualized, and multidisciplinary care [10,11,12,13,14].

The pathophysiology of COVID-19 remains a global conundrum, confounding neuromuscular, cardiovascular, metabolic, respiratory, and immune disorders on a very subject-dependent level [15,16]. The underlying systemic inflammation contributes to muscle atrophy indirectly (lung obstruction and forced inactivity) or directly (increased muscle wasting, necrosis, fibrosis and autophagy, and mitochondrial dysfunction), while viral antigen mimicry might cause muscle denervation in peripheral nerves. While physical activity (PA) has been identified as a predictive and protective factor against severe forms of COVID-19 [17,18,19,20], it has also been suggested as a key factor for rehabilitation [21,22,23], both for the acute and long-term consequences of this new global disease. According to a survey questioning the specific rehabilitation needs reported by post-COVID-19 patients themselves [24], exercise guidance appears to be a primary concern. The necessity of restoring their functional capacity seems particularly important for patients who have survived hospitalizations, since half of them present severely impaired physical functioning and a drastically reduced ability to perform daily activities after discharge [25]. Yet, not only for severe but also for mild forms of the disease, PA might help prevent the risk of complication, reduce the risk of long-lasting disability, and reintroduce patients to their own pre-COVID-19 life [26,27,28,29,30,31]. Although there is an impressive number of reviews or recommendation papers mentioning the interest in PA as part of COVID-19 rehabilitation, only a limited amount of clinical evidence is available. In addition, these studies seem heterogeneous in many aspects—such as in their designs, the characteristics of the populations, the disease severity, the duration of symptoms, the proposed programs, or the types of assessed outcomes—leading to complex interpretations of the results. In this context, a deeper review of the literature appeared highly interesting given the urgent need to provide evidence-based effective and relevant RPs for post-COVID-19 patients. Therefore, the present study aims at systematically analyzing the actual scientific literature that proposes a PA intervention for patients who contracted SARS-CoV-2.

## 2. Material & Methods

The study was conducted following the Preferred Reporting Items for Systematic Review and Meta-Analysis (PRISMA) guidelines [32]. All information about searches, selection process, eligibility criteria, data extraction process, risks of bias assessment, and data analysis were determined prior to the study and registered in the PROSPERO database (https://www.crd.york.ac.uk/prospero/, accessed on 19 May 2022) under the reference number CRD42022289219.

### 2.1. Literature Search Strategy and Study Eligibility

The literature search was conducted on PubMed (MEDLINE) electronic database. The search started on 1 August 2021 and the last re-run was performed on 31 December 2021. As the review aimed to investigate the effects of PA programs in patients that had previously contracted SARS-CoV-2, the following search equation was developed and used in the search strategy: (“COVID-19”[Title/Abstract] OR “SARS-CoV-2”[Title/Abstract]) AND (“training”[Title/Abstract] OR “exercise”[Title/Abstract] OR “physical activity”[Title/Abstract] OR “rehabilitation”[Title/Abstract]) AND (“humans”[Mesh]) AND (“patients”[Text Word]) NOT (“animals”[Title/Abstract] OR “rats”[Title/Abstract] OR “rodents”[Title/Abstract] OR “confinement”[Title] OR “containment”[Title] OR “lockdown”[Title] OR “obese”[Title] OR “obesity”[Title] OR “diabetes”[Title] OR “diabetic”[Title] OR “cancer”[Title] OR “review”[Title] OR “meta-analysis”[Title]). The references’ collection was performed using Zotero Software (5.0.21, CHNM, GMU, Fairfax, VA, USA). Two independent reviewers (M.B. and L.B.) systematically and separately performed the reviews and were blinded to each other’s decisions at all the stages of the screening and extraction process (literature search, selection against the eligibility criteria, data extraction/analyses, and evaluation of the risks of bias). Our reviewing team also included a third reviewer (D.T.) to discuss all the points of disagreements until a consensus was reached by all the members of the reviewing team.

### 2.2. General Inclusion Criteria

All the scientific papers published in English, French, German, or Spanish languages in peer-reviewed journals were eligible for inclusion in the review, according to the language skills of our reviewing team. All the clinical trials that investigated the effect of any kind of PA program among adults that had previously contracted SARS-CoV-2 were included in the systematic review. Acute, post-acute, and chronic-COVID-19 cases were eligible for inclusion. There were no restrictions on (1) the time of the beginning of the RP regarding the SARS-CoV-2 contraction, (2) the persistence of the symptoms, (3) the severity of the disease, (4) the gender, age, or comorbidities/pathologies, (5) the presence or absence of a comparator/control group, or (6) the type of outcomes/variables reported. All longitudinal studies including both quantitative and qualitative data were eligible for inclusion (randomized control trials, controlled trials, uncontrolled trials, prospective studies, or retrospective studies).

### 2.3. General Exclusion Criteria

Since our population of interest concerns post-COVID-19 patients, and since this virus appeared in November 2019, any article published before this date was systematically excluded. As the review aimed to assess the effect of an interventional PA program, all publications without original data or transversal studies were excluded, such as reviews, recommendation papers, meta-analyses, and letters to the editors without data. Case studies with less than 4 participants were also excluded. Clinical trials that explored the effect of RP without reporting any reference to PA approaches were also removed. The publications that did not enroll our population of interest were excluded (for instance children/adolescents <18 years or absence of COVID-19 infection).

### 2.4. Inclusion and Exclusion Criteria Specific to the Programs of PA

**Figure 1:**
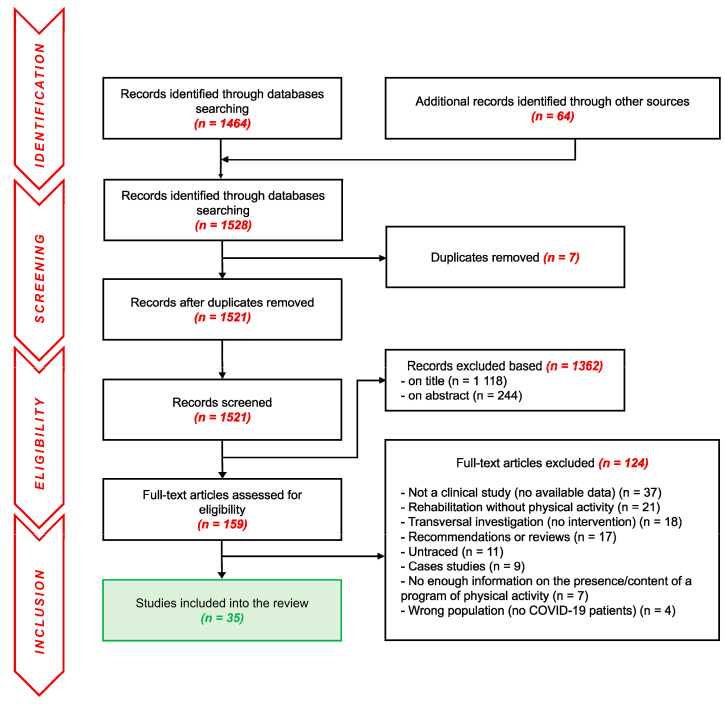
Flow diagram of the identification, screening, eligibility, and inclusion of the studies in the systematic review.

RPs performed in the post-COVID-19 population usually combine multidisciplinary strategies of rehabilitation, including (or not including) PA. As PA programs are almost solely proposed through these kinds of multidisciplinary approaches, the choice was made by the reviewing team to keep such multidisciplinary approaches eligible despite the risks of interactions between the different rehabilitative strategies. PA is defined here as “any bodily movement produced by skeletal muscles that requires energy expenditure” [33]. However, we considered PA as acceptable for the review only when an active mobilization of muscles was performed (even a very soft one such as postural changes, standing up, or walking). Any program that only implied passive mobilization (i.e., kinesiology or electrostimulation) was excluded. We consider here a very large definition of physical activity as any voluntary movements performed for recovery purposes—whatever the type of movement—as long as it was performed by the patient himself/herself. Given the very small number of publications even when considering all types of physical practices, the choice was collectively made to not provide any restrictions on the duration, modalities, intensities, frequencies, or volumes of physical activity. Both aerobic and resistance programs were accepted in the reviewing process. Programs of Traditional Chinese Medicine were excluded due to the complexity of the quantification of such alternative programs. More generally, all the publications without further details on the potential presence and/or content of a PA program integrated into their rehabilitation were excluded. The detailed flow chart of the selection process is presented in Figure 1.

### 2.5. Data Extraction and Synthesis

The main outcome of the present systematic review was to assess the evolution of functional capacity from baseline (before the program containing PA) to the last available follow-up. The secondary outcomes concerned the evolution of the respiratory function, quality of life, mental health, general state, biological parameters, adherence/tolerability, or even other specific parameters. Yet, as already detailed, all types of outcomes were eligible for inclusion as long as the effect of a PA program was explored. Data extraction was performed on two different spreadsheets using these different categories: (1) Reference, (2) Design of the study, (3) Severity of COVID-19 during the acute phase and duration of hospitalization/symptoms, (4) Start of the RP, (5) Rehabilitation interventions, (6) PA program, (7) Parameters assessed pre- and post-rehabilitation, (8) Effects of the RP on functional capacity, (9) Effects of the RP on respiratory function, (10) Effects of the RP on quality of life/mental health/general state, and (11) Additional results. For the main outcome, numerical results were extracted from the included articles while only global effects were reported for the secondary outcomes to remain concise and readable. A structured and organized extraction method was discussed between the two reviewers (M.B. and L.P.) prior to data extraction to homogenize the data synthesis between publications. Regarding missing data, inquiries were made to the authors of publications by email or on the ResearchGate platform. Without an answer within one month, missing data were considered unavailable. When any control or comparator group was available in the clinical trial, data were also extracted to be compared with the group of interest. A comparator or control group could be: COVID-19 patients without intervention, COVID-19 patients with the same intervention but different initial characteristics, patients with other respiratory issues but following the same RP, or any other group of patients that could provide relevant data for comparisons with the group of interest.

### 2.6. Criteria for Risks of Bias Assessment

**Figure 2:**
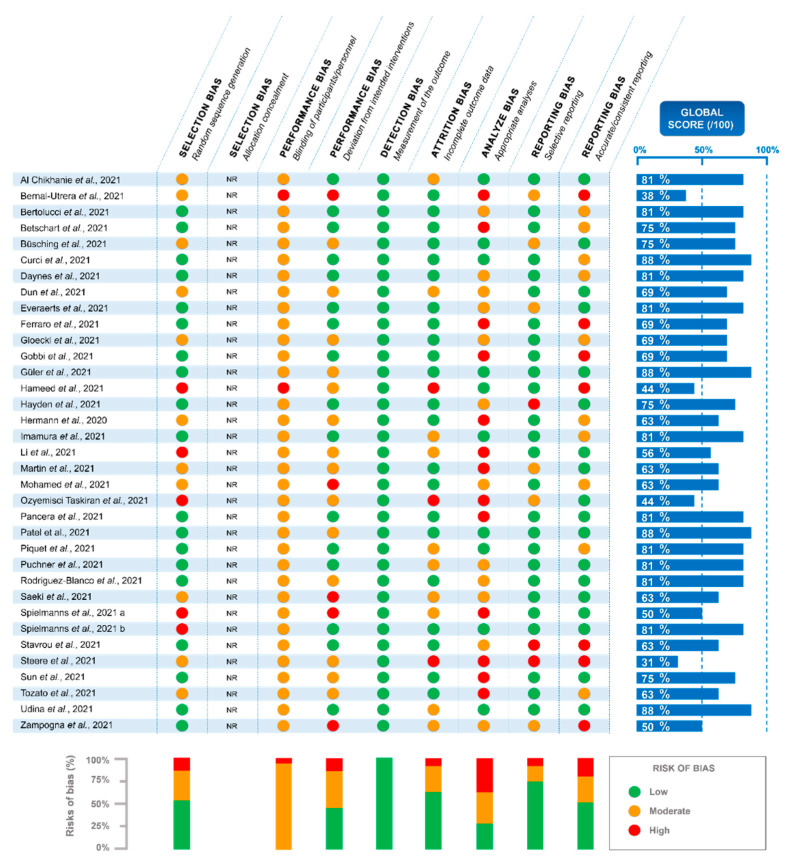
Analysis of the risks of bias in the studies included in the systematic review [35,36,37,38,39,40,41,42,43,44,45,46,47,48,49,50,51,52,53,54,55,56,57,58,59,60,61,62,63,64,65,66,67,68,69].

The assessment of the risk of bias was based on the Cochrane tool [34], and was divided into selection bias (random sequence generation and allocation concealment), performance bias (blinding of participants/personnel and deviation from intended interventions), detection bias (measurement of the outcome), attrition bias (incomplete outcome data), analysis bias (appropriate/inappropriate analysis), and reporting bias (selective reporting and accurate/consistent reporting). A global score per individual study was calculated as follows: low risk—2 points, moderate risk—1 point, and high risk—0 points for each bias category. A final score was then estimated as a percentage, which is illustrated by the blue bars in Figure 2.

The blue bars represent the global score per individual study calculated as follows: low risk—2 points, moderate risk—1 point, and high risk—0 points for each bias category. The final score was calculated as a percentage.

### 2.7. PA Program Scoring to Assess the Quality of the Description

**Figure 3:**
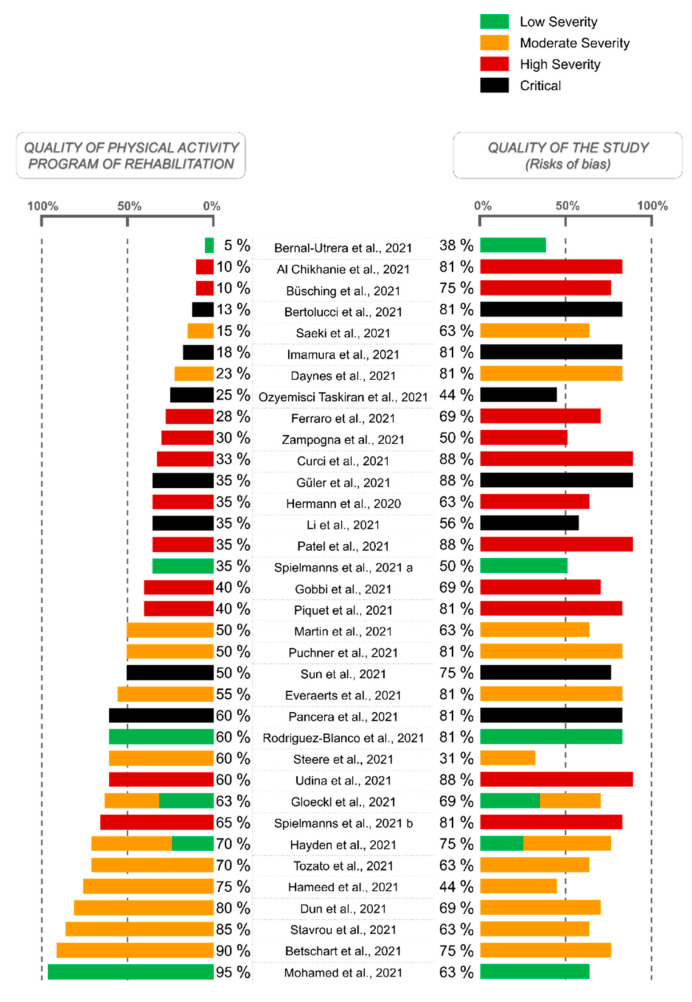
Physical activity program scoring to assess the quality of the description in relation to the COVID-19 severity presented by patients upon their entrance in the rehabilitation program [35,36,37,38,39,40,41,42,43,44,45,46,47,48,49,50,51,52,53,54,55,56,57,58,59,60,61,62,63,64,65,66,67,68,69].

A score representing the quality of the description of PA programs performed in the publications was calculated by our reviewing team (M.B. and L.P.), as illustrated in the left part of the histogram (Figure 3). As presented in detail in Table S1, a specific scale has been developed taking into account the level of details provided by each study concerning: (1) the activities, materials, and modalities of exercises (/5 points); (2) the frequency of the sessions, with indications per kind of activity performed/not performed (/4 points); (3) the volume of the sessions, i.e., total duration, number of repetitions, number of series, information on the recovery time, the warm-up and cool-down periods, and information per kind of activity performed/not performed (/5 points); and (4) the intensity of the sessions, i.e., use/non-use of different criteria such as Rate of Perceived Exertion (RPE) and dyspnea scales, estimated or real percentage of maximal heart rate (HR), estimated or real percentage of maximal work rate, and the percentage of Repetition Maxima (RM) (/6 points). Therefore, a score out of 20 points was calculated and finally expressed as a percentage representing the level of details provided by each article (0%—no any information; 100%—program described with maximum completeness enabling any health practitioner or researcher to implement it). This score does not assess the relevance of the program, but only the quality of its description and reproducibility.

The left bars of the histogram illustrate the quality of the physical activity program of rehabilitation and only assess the quality of its description and reproducibility, not its relevance. As detailed in the Methods section, a specific scale (Table S1) has been used to express the level of details provided by each article (0%—no information; 100%—program described with maximum completeness enabling any health practitioner or researcher to implement it).

The right bars of the histogram show the quality of the studies and perform the same indicatory function as the blue bars in Figure 2 (low risk—2 points, moderate risk—1 point, and high risk—0 points for each bias category; final score calculated as a percentage).

### 2.8. Estimation of COVID-19 Severity at the Beginning of Rehabilitation

Depending on the severity of the disease presented by the patient at the beginning of RP, a specific score was attributed by the reviewing team (M.B. and L.B.). This score was based on the medical assistance required by the patient (specific treatments or hospitalization are unnecessary, hospitalization is required, mechanical ventilation is required, and/or stay in an intensive care unit is required (ICU)), but also on other criteria such as the duration between the hospitalization and the recovery program, or the scores of their functional capacities at the beginning of the RP. Considering the heterogeneity of the study designs, types of groups, and patients’ characteristics, it appeared to be a complex task to define a specific scale such as those developed for the PA program. Accordingly, the score attributed to each study was deeply discussed between the members of the reviewing team until a consensus was reached (M.B., L.P., and D.T.). The severity was estimated as follows: 1–2 was considered critical, e.g., initially bedridden patients; 3–5 was considered high severity; 6–8 was considered moderate severity; and 9–10 was considered low severity (e.g., no hospitalization required or were capable independent living). The score of severity provided by Figure 3 was—as described earlier—estimated at the beginning of rehabilitation. This score had to be distinguished from the severity of the disease provided by Table S2, which was reported during the acute phase of the disease and thereby used another classification scheme (low severity—no hospitalization; moderate severity—hospitalization; high severity—hospitalization in ICU).

## 3. Results

### 3.1. Selection Process and Risks of Bias

The initial search yielded a total of 1464 original studies with 64 additional records identified from other sources. After the removal of the duplicates, 1118 records were excluded based on their titles and 244 on their abstracts. Among the 159 full-text articles assessed for eligibility, 124 were removed for different reasons further detailed in Figure 1. Finally, 35 studies [35,36,37,38,39,40,41,42,43,44,45,46,47,48,49,50,51,52,53,54,55,56,57,58,59,60,61,62,63,64,65,66,67,68,69] remained at the end of the study selection. As detailed in Figure 2, the global score for the risks of bias in-between studies were found to be heterogeneous, ranging from 31% [65] to 88% [40,47,57,68]. The higher risk of bias was observed for the ‘blinding of participants/personnel’ parameter assessed as ‘moderate risk’ for 94% [35,37,38,39,40,41,42,43,44,45,46,47,49,50,51,52,53,54,55,56,57,58,59,60,61,62,63,64,65,66,67,68,69] of the studies, and as ‘high risk’ for 6% of them [36,48]. The ‘analysis bias’ was the second most important bias, with 29% [35,39,40,47,48,51,57,58,63,68] of the studies considered ‘low risk’, 34% [37,41,42,43,45,49,54,59,60,61,64,69] ‘moderate risk’, and 37% [36,38,44,46,50,52,53,55,56,62,65,66,67] ‘high risk’. The lowest risk of bias was found for the detection bias with 100% [35,36,37,38,39,40,41,42,43,44,45,46,47,48,49,50,51,52,53,54,55,56,57,58,59,60,61,62,63,64,65,66,67,68,69] of the studies presenting a ‘low risk’ on this parameter. If no study was excluded based on the risk of bias assessment, the quality of the studies was then considered, as displayed in Figure 3.

### 3.2. Experimental Design and Constitutions of Groups

As reported in Table S2, 23 [36,37,38,41,43,44,45,46,48,49,53,54,55,59,60,61,63,64,65,66,67,68,69] studies were prospective, while 12 [35,39,40,42,47,50,51,52,56,57,58,62] were retrospective. Concerning the constitutions of the groups, 16 [36,37,38,40,41,43,47,51,52,57,58,59,64,66,68,69] studies were only constituted by a single group of COVID-19 patients during or the after acute phase (COV+), 5 [45,46,49,50,65] included several groups of COV+ patients, 4 [42,48,53,55] included a control group (CON) of COVID-19 patients during or after the acute phase but who did not perform the rehabilitation, 4 [35,39,62,63] compared the effects of an RP in COV+ and COV− (without previously contracting COVID-19 but performing a rehabilitation for other reasons) patients, 4 [44,56,61,67] were case studies (with more than 3 patients), 1 [60] was a controlled pilot study, and 1 [54] was a randomized control trial.

### 3.3. Characteristics and Severity of the Disease among COV+ Populations Performing the Rehabilitation

All the studies included both women and men, except for two [56,61], which included men exclusively (Table S2). No study included women exclusively. Among the groups of COVID-19 patients who performed an RP, the mean age of the population ranged from 39.4 [60] to 78.4 [68] years old, with 1 [60] study including a group younger than 40 years old, 19 [36,41,42,43,45,47,48,49,51,54,56,59,61,65,67,68] groups aged between 40 and 60 years old, and 23 [35,37,38,39,40,44,45,46,48,50,52,53,55,57,58,62,63,64,66,68,69] groups of patients older than 60 years old. The mean body mass index (BMI) was under 25 kg/m^2^ in 16% [42,45,46,52,54,55,56] of the included studies, between 25 and 30 kg/m^2^ in 49% [35,39,40,43,45,46,47,48,49,50,51,53,58,59,61,62,63,69] of the studies, higher than 30 kg/m^2^ in 7% [48,49,64], and not reported for the remaining 28% [36,37,38,41,44,57,60,65,66,67,68]. The population presented comorbidities in 79% [35,37,38,39,40,41,42,43,44,45,47,48,50,51,52,55,56,57,58,59,61,62,63,64,65,66,67,68,69] of the studies (7% [36,54,60] without comorbidity, and 14% [46,49,53] not reported). During the acute phase of the disease, 29% [35,37,40,51,52,53,55,56,57,61] of the studies included patients presenting high severity, 54% [39,41,42,43,44,46,47,48,49,50,58,59,63,64,65,66,67,68,69] moderate-to-high severity, 3% [45] low-to-high severity, 6% [38,54] moderate severity, 3% [62] low-to-moderate severity, and 6% [36,60] low severity (Table S2) (high—hospitalization in ICU; moderate—hospitalization; low—no hospitalization).

### 3.4. Rehabilitation Interventions

The RP was performed directly in the hospital/medical facility for 74% [35,37,38,39,40,41,43,44,45,46,47,49,50,51,52,55,56,57,58,59,62,63,66,67,68,69] of the studies, in telerehabilitation for 20% [36,48,53,54,60,64,65] of the studies, and both for the remaining 6% [42,61] (Table S2). The start of the RP ranged from immediately during the acute phase [36,56,60,62,66] of the disease to 4.7 months after the disease [49]. In 31 [35,36,37,38,39,40,41,42,44,45,46,47,48,49,50,51,52,54,55,56,57,58,59,62,63,64,65,66,67,68,69] studies, the PA program was only a part of a multidisciplinary program combined with other approaches such as respiratory exercises, therapeutic education, psychological counseling, nutritional support, or kinesiology. Only 4 [43,53,60,61] studies performed a PA program alone (without another kind of RP).

### 3.5. PA Programs

A total of 6% [42,54] of the studies exclusively performed an aerobic program, 9% [48,60,66] of the studies exclusively performed a resistance program, and 77% [35,37,38,39,40,41,43,44,45,46,47,49,50,51,53,56,57,58,59,61,62,63,64,65,67,68,69] of the studies combined aerobic and resistance modalities (unclear or not applicable for 9% [36,52,55]). Only six [38,42,49,54,64,67] studies provided all the details on the type, frequency, volume, and intensity of the PA program (Table S2). For the 29 [35,36,37,39,40,41,43,44,45,46,47,48,50,51,52,53,55,56,57,58,59,60,61,62,63,65,66,68,69] other studies, at least one of these parameters was not reported. As displayed in Figure 3, the quality of the description concerning the PA program was quite low, with 21 [35,36,37,39,40,41,44,46,47,50,51,52,53,55,57,58,59,61,62,66,69] out of the 35 included studies presenting a score of 50% or below. When combined with the severity of the disease upon admission to the rehabilitation program, it appears that the studies that included the most severe forms are relatively the same as the studies with the poorer PA description (Figure 3). However, no relationship between the quality of the studies (the risk of bias assessment) and both the COVID-19 severity and the quality of the PA programs seems to appear.

### 3.6. Parameters Assessed and Methods Used

All but four studies [47,54,62,65] reported results on functional capacity (Tables S2 and S3). To assess functional capacity, 51% [35,38,39,40,42,43,44,45,49,50,59,60,61,63,64,67,68,69] of them performed a 6-minute walk test (6 MWT), 29% [35,43,44,45,46,55,58,61,64,67] a handgrip test, 17% [45,48,53,58,60,64] a sit-to-stand test, 14% [44,51,56,68,69] a short physical performance battery, 14% [51,52,55,56,61] reported the patients’ muscle strength using the Medical Research Council scoring criteria (scored 1–60), 9% [35,43,45] assessed the quadriceps’ force, 6% [41,45] performed the incremental and/or endurance shuttle walking test, 6% [52,57] a walking distance test, 3% [67] a 1-RM, 3% [35] a Tinetti test, 3% [43] a cardiopulmonary exercise test (CPET), 3% [44] a 10 MWT, 3% [46] a time-up-to-go test, 3% [48] a 2-min step test, 3% [55] assessed ranges of motion, 3% [64] estimated the metabolic equivalent from the 6 MWT, 3% [52] used physical function and mobility scores, 3% [61] measured gait speed, 3% [68] assessed the ability to perform an unassisted gait, 3% [36] only reported the patients’ subjective impression, 3% [37] used electromyography to assess the peripheral nervous system, 3% [66] observed the ability to perform simple gestures, and 3% [68] performed a standing balance (on a single leg—maintaining 10 s). While no study performed a sub-maximal or maximal incremental test to assess VO_2peak_ in patients, two [43,64] of them estimated this parameter from the CPET [43] or the 6 MWT [64]. Moreover, at rest, during and/or after a physical exercise test, 14% [45,53,58,64,67] of the studies measured HR, 11% [40,58,60,67] assessed the RPE using the Borg scale, 3% [64] measured systolic and diastolic blood pressure, 3% [64] the lower extremity fatigue (Borg scale), and 3% [67] calculated the HR multiplied by the systolic blood pressure. Patients’ respiratory function was evaluated in 66% [35,36,37,40,41,43,45,46,47,48,49,50,51,52,53,56,57,58,59,61,64,66,67] of the studies, quality of life/mental health/general state in 80% [35,36,37,38,39,40,41,43,44,45,47,49,50,51,52,54,55,56,57,58,59,61,62,63,64,66,68,69], biological parameters in 23% [40,42,45,46,49,54,64,66], and adherence/tolerability was examined in 77% [35,36,37,38,40,41,42,43,44,45,48,49,50,51,52,53,54,55,56,58,59,60,62,64,66,68,69] of the studies.

### 3.7. Effects of the RP

Among the 29 [35,38,39,40,41,42,43,44,45,46,48,49,50,51,52,53,55,56,57,58,59,60,61,63,64,66,67,68,69] studies with statistical analyses assessing the evolution of functional capacity, 97% [35,38,39,40,41,42,43,44,45,46,48,49,50,51,52,53,56,57,58,59,60,61,63,64,66,67,68,69] reported a significant improvement in at least one parameter of functional activity (Table S3). The entirety of the 18 [35,38,39,40,42,43,44,45,49,50,59,60,61,63,64,67,68,69] studies that performed statistical analyses of the functional capacity by using the 6 MWT found a significantly higher walked-distance after the rehabilitation. Among the 18 [35,40,41,43,45,46,48,49,51,52,53,56,58,59,61,64,66,67] studies statistically assessing the effects on the respiratory function, 72% [35,40,41,43,45,46,48,49,52,58,59,64,66] demonstrated a significant effect on the respiratory system. Ninety-six percent [35,37,38,39,40,41,43,44,45,47,49,50,51,52,54,56,57,58,59,61,63,64,66,68,69] of the 26 [35,37,38,39,40,41,43,44,45,47,49,50,51,52,54,55,56,57,58,59,61,63,64,66,68,69] studies that statistically investigated the effects on the quality of life, mental health, and general state observed a positive effect of the RP (Table S3). Given the high heterogeneity of the parameters assessed and the statistics performed between the different studies included, a further general overview of these results was complex. Yet, the case-by-case details are provided in Tables S2 and S3.

## 4. Discussion

Since PA may be of particular interest for the treatment of post-COVID-19 patients, there is an urgent need to establish appropriate RPs and to better evaluate their efficacy. Therefore, the present study proposed a systematic analysis of the scientific literature that evaluates the effect of PA programs in COVID-19 patients who contracted the SARS-CoV-2.

First, about one-third of the included studies were retrospective and did not initially plan to specifically evaluate the effects of a PA program. In addition, our results highlight a high methodological heterogeneity regarding the experimental designs with both controlled and uncontrolled studies and different approaches for the constitution of groups (Table S2). Out of the 35 [35,36,37,38,39,40,41,42,43,44,45,46,47,48,49,50,51,52,53,54,55,56,57,58,59,60,61,62,63,64,65,66,67,68,69] included studies, only one randomized controlled trial [54] was found. The systematic review of the literature also emphasized a large proportion of case studies (*n* = 13 [29,30,44,56,61,67,70,71,72,73,74,75,76]), leading us to only retain studies that enrolled a minimum number of four participants (*n* = 4 [44,56,61,67] to be finally included). This large number of retrospective studies, uncontrolled studies, and case studies probably reflects the emergency of the pandemic. In this context, well-designed research protocols were probably difficult to establish and to conduct in such a short period and in a situation of sanitary crisis where the first priority was to receive and save patients with severe forms of illness. Logically, many studies based their RP on pre-existing pulmonary RPs since respiratory impairments remained among the most serious and persistent symptoms among COVID-19 patients [77]. Yet, the three studies [35,39,63] that conducted the same RP with PA in a group of patients with other affections including lung diseases (Chronic Obstructive Pulmonary Disease, pneumonia, infectious pulmonary disease, and lung cancer) compared with COVID-19 patients observed different results: COVID-19 patients would tend to recover more quickly than the patients with other pulmonary affections. Therefore, this observation clearly calls for the development of an RP specific to SARS-CoV-2 infection. Indeed, although major defects mainly concern the respiratory system, specific approaches to different body systems should be conducted since many implications can be observed, such as neuromuscular, cardiovascular, digestive, nervous, metabolic, immune, inflammatory, skin, taste, smell, or sleep implications [3,4,78].

A second observation was that the studies mainly included overweight, aged patients with various comorbidities, who are known to represent important risk factors for severe forms of COVID-19. Despite a large age difference between the included studies, with the patients’ mean ages ranging from 39.4 [60] to 78.4 [68] years old, none of them have been performed on young adults without comorbidities (<35 years old). This might also explain why a high level of disease severity was observed in the populations’ characteristics of the included studies (Table S2). While the hospitalization rate and severe-to-critical forms amounted to around 15-to-20% (before the large-scale vaccination) [27], 94% [35,37,38,39,40,41,42,43,44,45,46,47,48,49,50,51,52,53,54,55,56,57,58,59,61,62,63,64,65,66,67,68,69] of the selected studies enrolled patients who required hospitalization or intensive care (Table S2); therefore, this proportion is not truly representative of the general population contracting the disease. Indeed, even a low or moderate severity of COVID-19 can imply long-term sequels [8]. Potentially linked with the severity of the disease, the present systematic review also revealed that three-quarters of the programs were performed directly at the hospital or in specialized medical facilities [35,37,38,39,40,41,43,44,45,46,47,49,50,51,52,55,56,57,58,59,62,63,66,67,68,69]. Indeed, Figure 3 clearly showed that only very few studies started their PA intervention in patients with a low degree of severity [36,45,49,54,60,62]. As pointed out by Belli et al. [25], it also appears to be important to propose an early referral to rehabilitative intervention options in the post-hospitalization phase, even after early mobilization and bedside physiotherapy. However, the review also showed that some programs were individually performed, home-based, and/or were forms of telerehabilitation, with remote support and assistance [36,48,53,54,60,64,65]. Although telerehabilitation programs represent the minority of the studies, they might be nonetheless essential. Indeed, with the massive vaccination achieved in the last months, less severe forms seem to occur in proportion to the number of positive cases [79]. Yet, at the same time, the contamination rates have reached record levels in these recent months [1]. Thus, mild COVID-19 cases might represent even larger numbers in the months or years to come, for whom a recovery program using PA might present important direct and long-term benefits. In addition, PA has been suggested as a “coadjuvant treatment” to COVID-19 vaccination to prevent an upstream risk of infection via a potential improvement in immune responses [80,81]. While vaccination seems to have minimal effects on physiological responses to exercise in healthy individuals [82], future studies investigating the interaction between recovery, PA rehabilitation, and vaccines in post-COVID-19 patients should be relevant. Concerning the initiation of the rehabilitation, it was found highly variable in-between studies, ranging from ‘immediate’ during the acute phase [36,56,60,62,66] to 4.7 months after the infection [49]. This might also be quite dependent on the severity of the disease and on the time needed to be able to stand, for the most severe forms. 

In addition, Figure 3 also seems to show that the studies with the higher levels of severity are also those with the least described PA programs. Once more, we did not assess the relevance of the PA program, but only the quality of its description and reproducibility. We suggest that the severity of the disease might be related to a poor description/reproducibility of the program because of different reasons, such as the heterogeneity of both the initial and evolutive functional capacity in patients with severe forms of COVID-19, the potential inability to stand at the beginning of the program, the higher importance of the respiratory rehabilitation, etc. Yet, even in less severe forms, the quality of the PA programs remains moderate. We suggest that this might be due to the retrospective nature of several studies, which is related to the emergency of the health crisis, and that possibly results in the absence of a staff specialized in PA therapy. In addition to the lack of description, the programs of PA appear highly heterogeneous in terms of their total duration, modalities, exercises, volume, frequency, and intensity (Table S2), making any comparison of the studies’ results very complex (Table S3). Another important factor that considerably reduces the ability to compare the results in-between the studies is the high heterogeneity of the methods used to evaluate the programs effects. Indeed, the most frequently performed test of functional capacity was the 6 MWT; however, it was only conducted by half of the studies [35,38,39,40,42,43,44,45,49,50,59,60,61,63,64,67,68,69]. All the other types of measurements were infrequently used (less than one in three studies). More than 30 different methods were used to assess functional capacity, thereby demonstrating a wide variety and heterogeneity. Due to the disparity of many of the criteria (experimental designs, characteristics of the populations, PA programs, and the methods used), the results of the different studies could not be compared with each other using meta-analytical approaches. Although assessed using different methods, functional capacity, respiratory function, and quality of life/mental health/general state were largely considered, assessed by 89% [35,36,37,38,39,40,41,42,43,44,45,46,48,49,50,51,52,53,55,56,57,58,59,60,61,63,64,66,67,68,69], 66% [35,36,37,40,41,43,45,46,47,48,49,50,51,52,53,56,57,58,59,61,64,66,67], and 80% [35,36,37,38,39,40,41,43,44,45,47,49,50,51,52,54,55,56,57,58,59,61,62,63,64,66,68,69] of the included studies, respectively. Since these three parameters are the most frequently affected and disabling ones for post-COVID-19 patients [3,4,8,9,10], they logically appear to be the most frequently investigated according to this research. Among the studies with statistical analyses, the systematic review showed highly beneficial effects of the RPs including PA. Almost all the studies found significant improvements in functional capacity (97% [35,38,39,40,41,42,43,44,45,46,48,49,50,51,52,53,56,57,58,59,60,61,63,64,66,67,68,69]) and 100% [35,38,39,40,42,43,44,45,49,50,59,60,61,63,64,67,68,69] of the studies observed significant enhancements when using the 6 MWT. Although a little less extensive, 72% [35,40,41,43,45,46,48,49,52,58,59,64,66] of the studies revealed significant improvements in respiratory function after a RP including PA. Another very important parameter for the patients’ well-being concerns their quality of life, mental health, and general state, which has been found to be significantly improved in 96% [35,37,38,39,40,41,43,44,45,47,49,50,51,52,54,56,57,58,59,61,63,64,66,68,69] of the studies with available statistics. Thus, the systematic review clearly showed an unequivocal efficacy of RP including PA for both the functional capacity and quality of life of COVID-19 patients, while this efficacy appears a little less clearly observed concerning respiratory function, which might be explained by different initial impairments. Although the pathophysiology appears to be multifactorial, post-COVID-19 syndromes are often a consequence of neuromuscular and cardiovascular abnormalities associated with persistent unresolved inflammation and/or long-term tissue damage [83], resulting in a high impact on both a patient’s physical and psychological health. Moreover, if untreated, the hyperinflammatory component can induce the appearance of additional metabolic disorders, such as type 2 diabetes, with a reported co-emergence of clinical depression (if not already present) [16]. Thus, the beneficial effect of PA is plausible since it improves immune dysfunction through the release of anti-inflammatory myokines, the stimulation of lymphovascular circulation, and lymphocyte mobilization, along with strengthening weakened muscle structure and reestablishing neuromuscular activation [15,26].

The present systematic review presents some limitations. First, 83% [35,36,37,38,39,40,41,43,44,45,46,47,49,50,51,52,56,57,58,59,61,62,63,64,65,66,67,68,69] of the included studies did not include a control group of COVID-19 patients without a PA intervention. In this context, it remains difficult to assess whether the improvements observed were due to the RP or to the natural recovery process. Secondly, almost all the analyzed studies included PA in a multidisciplinary approach; therefore, it was impossible to distinguish the exact effects of PA itself from the role played by the other approaches (such as respiratory exercises, therapeutic education, psychological and nutritional support, or kinesiology). Yet, if these multidisciplinary approaches can be criticized from a methodological perspective, their use is justified and essential for the benefit of patients from an ethical point of view. In addition, the included studies proposed diverse types of PA programs and enrolled groups of patients with various comorbidities, suffering from different COVID-19 severities, and presenting with a large range of ages. This important heterogeneity, both in terms of population and RPs, highly limited the comparison between studies and forced us to make only general conclusions. Thirdly, although the risks of bias assessment showed a relatively great quality of the included studies, some biases remained more important than others and should be mentioned. Statistical analyses were not performed, or not in a relevant way, for 71% [36,37,38,41,42,43,44,45,46,49,50,52,53,54,55,56,59,60,61,62,64,65,66,67,69] of the studies (Figure 2 and Figure 3). The types of outcomes assessed here could not be blinded from the personnel and participants most of the time, which induced a quite important potential performance bias. In addition, Figure 2 showed a relatively important level of inaccuracy/inconsistency in the result-reporting of some of the included studies. Moreover, only one database was consulted for the literature search. Although this represents an important limitation, this decision was made collectively in the context of a health emergency regarding the urgent need to propose a first overview of PA’s effects on COVID-19 rehabilitation. Based on the peer review process, PubMed (MEDLINE) is also recognized to achieve a high exhaustivity and ensure the quality of data. Lastly, the potential impacts of the vaccination or the different SARS-CoV-2 variants have never been taken into consideration or even mentioned in the included studies.

## 5. Conclusions

This systematic review unequivocally showed that RPs containing PA result in improvements in functional capacity, respiratory function, and the quality of life. These health benefits were even observed for a very low intensity of physical activity, such as minor muscle re-mobilization (i.e., postural changes and/or walking a few meters, completed twice a day) for the most affected patients, and for low volumes such as 120 min per week of aerobic exercise for mild or moderate cases. However, the present results were only obtained from data on aged people with various comorbidities and presenting moderate-to-severe forms of the COVID-19 disease, but interestingly among the frailest patients at a high risk of mortality, thereby demonstrating its feasibility and efficacy. The PA programs were poorly described, heterogeneous, and the PA was usually only a part of a multidisciplinary RP. One-third of the studies were uncontrolled, making the observed improvements questionable, possibly due to a natural recovery. One-third of the studies were retrospective and thereby not initially designed for a standardized program of PA. Regarding all these observations, the systematic review suggests the urgent need to perform prospective controlled clinical trials with well-designed programs of PA, not only in aged patients with comorbidities and severe forms of illness, but also in young and/or initially healthy people presenting mild illness.

## Supplementary Materials

The following supporting information can be downloaded at: https://www.mdpi.com/article/10.3390/ijerph19159025/s1, Table S1: Level of details provided by the included studies on the program of physical activity; Table S2: Designs of the studies, characteristics of the population, description of the intervention, and method used in the included studies; Table S3: Effects of the rehabilitation program on function capacity, respiratory function, and quality of life in post-COVID-19 patients.
