# Supplementary material for: Systematic Review of COVID-19-Related Physical Activity-Based Rehabilitations: Benefits to Be Confirmed by More Robust Methodological Approaches
Source: Int J Environ Res Public Health. 2022 Jul 25;19(15):9025. doi: 10.3390/ijerph19159025 (PMC9331032; doi:10.3390/ijerph19159025)
Supplement: Supplementary file 1 [file ijerph-19-09025-s001.zip › ijerph-1757327-supplementary.pdf]

Supplementary Table S1: Level of details provided by the included studies on the program of physical activity.

Types of Exercises				Frequency		Volume of sessions				Intensity of sessions			
NR			**0pt**	NR	**0pt**	NR			**0pt**	NR			**0pt**
						For all modalities only			**1pt**				
**AEROBIC**		**RESISTANCE**				**AEROBIC**		**RESISTANCE**		**AEROBIC**		**RESISTANCE**	
		Worked muscles *(e.g. lower limbs, abdominal muscles)*	**2pt**			Total duration	**4pt**	Total duration	**1pt**	RPE and/or Dyspnea	**1pt**		
Activity only *(e.g. Walking)*	**2pt**	Material used *(e.g. elastics, machines, free weight)*	**2pt**	Frequency for all modalities	**2pt**			Number of repetitions	**2pt**	$\%HR_{max}E$ or $\%WR_{max}E$	**2pt**	RPE and/or Dyspnea	**2pt**
Activity + Material/place *(e.g. Walking on a treadmill/on a plane surface)*	**3pt**	Type of exercises *(e.g. squat, bench press)*	**3pt**					Number of repetitions + series	**3pt**	$\%HR_{max}$ Real or $\%WR_{max}$ Real (from a previous test)	**3pt**		
Activity + Modality *(e.g. HIIT or MICT walking)*	**4pt**							Number of repetitions + series + number of exercises and/or total duration	**4pt**			% of RM	**4pt**
Activity + material/place + modality *(e.g. MICT walking on a treadmill)*	**5pt**	Worked muscles and/or type of exercises + Material used *(e.g. bench press using a machine)*	**5pt**			Total duration + Details on warm-up and/or cooling-down	**5pt**	Number of repetitions + series + number of exercises and/or total duration + Recovery time	**5pt**	RPE and/or Dyspnea + $\%HR_{max}E$ or %PowerE	**5pt**		
				Frequency for each modality	**4pt**	Details for each modality + all modalities (bonus point)			**0.5pt**	RPE and/or Dyspnea + $\%HR_{max}$ Real or $\%WR_{max}$ Real (from a previous test)	**6pt**	RPE and/or Dyspnea + % of RM	**6pt**
/5 points (mean between aerobic and resistance)				/4 points		/5 points (mean between aerobic and resistance)				/6 points (mean between aerobic and resistance)			

The higher mark on each subscore is retained for the total score calculation (subscores not cumulative). HIIT: High Intensity Interval Training, HR_{max}: maximal Heart Rate, $HR_{max}E$: Estimated maximal Heart Rate, MICT: Moderate Intensity Continuous Training, NR: Not Reported, pt: points, RM: Repetition Maxima, RPE: Rated Perceived Exertion, WR_{max}: maximal Work Rate, $WR_{max}E$: Estimated maximal Work Rate.

Supplementary Table S2: Designs of the studies, characteristics of the population, description of the intervention, and method used in the included studies.

Reference	Design of the study	Characteristics of the population (n; age; BMI) and comorbidities	Severity of COVID-19 during the acute phase and duration of hospitalization/symptoms (low severity: no hospitalization; moderate severity: hospitalization; high severity: hospitalization and intensive care)	Start of the rehabilitation program	Rehabilitation interventions	Physical activity program	Parameters assessed pre- and post-rehabilitation (detailed only when available results
Al Chikhanie et al., 2021 [35]	Retrospective<br>Two groups	COV+ (post-ICU):<br>n=21 (14f, 7m);<br>70.9±10.6 yo;<br>26.9±5.4 kg/m²<br>At least 1 comorbidity/subject among its: CVD, DM, cancer or obesity<br><br>COV- (post-ICU):<br>n=21 (13f, 8m);<br>69.1±9.4 yo; 24.7±7.2 kg/m²<br>COPD (n=15), Stroke, cirrhosis or CVD (n=6)	**High**<br>ICU:<br>- COV+ (n=21): 23.4±8.5 days<br>- COV- (n=21): 16.2±26.9 days<br>Intubation:<br>- COV+ (n=16): 22.3±5.7 days<br>- COV- (n=6): 1.6±2.9 days<br><br>Groups comparison: significant differences for number of intubated ($p<0.05$) and days intubated ($p<0.05$)	Duration between extubation and RP: range: 3-45 days	In hospital (rehabilitation center)<br><br>Physical activity, respiratory exercises<br><br>Duration:<br>- COV+: 27.6±14.2 days<br>- COV-: 29.9±17.3 days	Modality: aerobic<br>Type: walking, cycling<br>Frequency: NR<br>Volume: NR<br>Intensity: NR<br><br>Modality: resistance, balance<br>Type: gymnastic<br>Frequency: NR<br>Volume: NR<br>Intensity: NR	<u>Functional capacity:</u><br>6MWT, Handgrip, Quadriceps isometric, Balance (Tinetti test)<br><br><u>Respiratory function:</u><br>FEV1 and FVC (method NR), Need for oxygen therapy, PI_{max} and PE_{max} (method NR), Dyspnea post-6MWT (Borg scale, probably 0-10), SaO_{2min} during 6MWT<br><br><u>Quality of life, mental health, and general state:</u><br>QoL (Saint George respiratory questionnaire), Anxiety and depression (HADS), Post-traumatic stress (Post-traumatic stress disorder checklist scale), Fatigue (Pichot questionnaire)
Bernal-Utrera et al., 2021 [36]	Prospective Controlled trial, initially two interventional groups and one control group, but only results from the interventional groups	COV+:<br>n=25 (18f, 7m);<br>41.4±13.4 yo; NR<br>No comorbidity<br><br>Some patients performed the toning exercises program and some patients performed respiratory program (2 groups), but unseparated results and no further details on how many patients performed each program - Protocol article (Gonzalez-Gerez et al. 2020)	**Low**<br>No hospitalization/very mild symptoms	During the acute phase (time period that patients diagnosed with COVID-19 were obligated to stay at home)	Telerehabilitation (no-supervised)<br><br>Physical activity, respiratory exercises<br><br>Duration: 14 days, but higher duration for some patients who needed to continue with the intervention	Modality: NR<br>Type: 10 toning exercises (no further information)<br>Frequency: NR<br>Volume: NR<br>Intensity: NR	<u>Functional capacity:</u><br>6MWT (assessments every day by the patient himself at home), 30-s STST, RPE during 6MWT and 30-s STST (Borg scale, type NR)<br>Semi-structured interviews<br><br><u>Respiratory function:</u><br>FEV1 and PEF (spirometry, assessments every day by the patient himself), Dyspnea post-6MWT (Multidimensional Dyspnea-12 questionnaire - Spanish version)<br>Semi-structured interviews<br><br><u>Quality of life, mental health, and general state:</u><br>Fatigue (Fatigue VAS)<br>Semi-structured interviews
Bertolucci et al., 2021 [37]	Prospective<br>Single group	COV+:<br>n=39 (15f, 24m);<br>67.8±10.8 yo; NR<br>Obesity (n=14), DM (n=10), No comorbity (n=14)<br>CIRS: median: 1 [IQR: 0, 3]	**High**<br>ICU (n=32) or COVID unit hospital (n=7): 46.4±20.9 days<br>Intubation (n=23): range: 4-36 days<br><br>Symptoms at admission to RP:<br>Bacterial superinfection (n=23), Prone ventilation (n=17)	Immediately after hospital discharge, 82 and 137 days after hospital discharge for 2 patients	In hospital (rehabilitation unit)<br><br>Physical activity, respiratory exercises, swallowing rehabilitation<br><br>Duration: 25.5±16.3 days	Modality: aerobic<br>Type: walking (IT or continuous)<br>Frequency: NR<br>Volume: NR<br>Intensity: NR<br><br>Modality: resistance<br>Type: NR<br>Frequency: NR<br>Volume: NR<br>Intensity: NR<br><br>Modality: coordination and posturology<br>Type: active postural changes, readjustment of postural reflexes, coordination exercises for trunk control, recovery of standing position	<u>Functional capacity:</u><br>Peripheral nervous system impairment (electromyography)<br><br><u>Respiratory function:</u><br>Need for tracheostomy, Need for oxygen therapy, PaO_2 and FiO_2 (method NR)<br><br><u>Quality of life, mental health, and general state:</u><br>Mental confusion (method NR), Disability level (BI), Ambulation capacity (FAC)<br><br><u>Other:</u><br>Corticosteroids intake, Antipsychotic intake, Dysphagia (Need for enteral alimentation), Rectal colonization by multidrug resistant Enterobacteriaceae

						Frequency: NR<br>Volume: NR<br>Intensity: N/A	
Betschart et al., 2021 [38]	Prospective Single group	COV+:<br>n=12 (4f, 8m); 61 yo (range: 26-84); NR<br>CVD (n=6), CKD (n=5), HT (n=3), Cancer (n=3), Chronic pulmonary disease (n=2), DM (n=1), Overweight/obesity (n=1), Polyneutropathia (n=1), Other disease (n=2)	**Moderate**<br>Hospitalization: 11 days (range: 3-24)<br><br>Severity of pneumonia: Mild (n=1), Moderate (n=8), Severe (n=2), Critical (n=1)	Duration between COVID-19 diagnosis and RP: 41.5 days (range: 21-73)<br>(One of the selection criteria: at least 14 days after confirmed diagnosis of COVID-19 and at least 4 days without COVID-related symptoms)	In hospital<br><br>Physical activity, education<br><br>Duration: 4-13 wk	Modality: aerobic<br>Type: cycling (HIIT or MICT, probably on ergometer)<br>Frequency: 2x/wk<br>Volume: 30min<br>Intensity: REP=4-6 (Borg scale 0-10)<br>- HIIT: 4min 15%WR_{peak} (warm-up), [4 x 4min 50%WR_{peak} (RPE=4-6, Borg scale 0-10) + 3 x 3min 20-30%WR_{peak}], 3min 15%WR_{peak} (cooling-down)<br>- MICT: 20-30%WR_{peak}<br>(WR_{peak} assessed using the incremental ultra-short maximal steep ramp test)<br><br>Modality: resistance<br>Type: chest press, low row, back extension, leg abductor, leg curl, leg press<br>Frequency: 2x/wk<br>Volume: 3sets x 10-12rep (30-40min)<br>Intensity: 50-85%RM (RPE=4-6, Borg scale 0-10)	Functional capacity:<br>6MWT<br><br>Quality of life, mental health, and general state:<br>QoL (EQ5D 5 levels, EQ5D VAS), Perception of COVID-19 specific limitations in daily life (Post-COVID Functionality score)<br><br>Other:<br>Adherence and tolerability of the program
Büsching et al., 2021 [39]	Retrospective Controlled trial, two groups	COV+ (with pneumonia):<br>n=51 (13f, 38m); 65.8±11.7 yo; 27.3±4.9 kg/m²<br>HT (n=30), Acute respiratory distress syndrome (n=26), CVD (n=8), COPD (n=2)<br>CIRS: 17.7±11.3<br><br>COV- (with pneumonia):<br>n=51 (28f, 23m); 69.8±9.6 yo; 26.1±6.5 kg/m²<br>COPD (n=25), HT (n=19), CVD (n=10), Acute respiratory distress syndrome (n=3)<br>CIRS: 13.5±5.9<br><br>Groups comparison: gender repartition ($p<0.01$), age ($p<0.05$), comorbidities ($p<0.001$), CIRS ($p<0.05$)	**Moderate-to-High**<br>ICU (COV+: n=30; COV-: n=7)<br>Groups comparison: COV+ > COV-<br>Intubation:<br>- COV+ (n=27): 13±7 days<br>- COV- (n=6): 10±8 days	Immediately after discharge hospital	In hospital<br><br>Physical activity, education (minimum 540 min), respiratory exercises, relaxation techniques, and if necessary, psychological counseling, speech, nutritional and occupational therapy, social services<br><br>Duration:<br>- COV+: 22±6 days<br>- CON: 20±5 days	Modality: aerobic<br>Type: cycling, guided walking<br>Frequency: NR<br>Volume: NR<br>Intensity: NR<br><br>Modality: resistance<br>Type: machines, free weight, elastic bands<br>Frequency: NR<br>Volume: NR<br>Intensity: NR	Functional capacity:<br>6MWT<br><br>Quality of life, mental health, and general state:<br>Disability level (Chronic respiratory questionnaire, FIM)
Curci et al., 2021 [40]	Retrospective Single group	COV+:<br>n=41 (16f, 25m); 72.2±11.1 yo; 25.7±3.7 kg/m²<br>HT (n=26), DM2	**High**<br>ICU: 18.0±8.7 days<br><br>Respiratory status at admission: No respiratory support (n=7), Nasal	Immediately post-ICU	In hospital (COVID-19 rehabilitation unit)	Modality: aerobic<br>Type: walking<br>Frequency: NR<br>Volume: NR<br>Intensity: NR	Functional capacity:<br>6MWT, RPE probably post-6MWT (Borg scale, probably 6-20)<br><br>Respiratory function:<br>Dyspnea in daily life activities (mMRC Dyspnea scale), Respiratory supports

		(n=11), Malignancy (n=10), Pulmonary embolism (n=8), Dyslipidemia (n=7), Myocardial infarction (n=6), Arrythmia (n=5), Stroke (n=5), HT (n=3), COPD (n=3), Hepatitis (n=3), Hypothyroidism (n=3), CKD (n=3), No comorbidity (n=3)	cannula (n=18), Venturi mask (n=8), Oxygen mask (n=5), Non-rebreather mask (n=3)		Physical activity, respiratory exercises, kinesiology<br><br>Duration: 32.0±9.1 days	Modality: resistance<br>Type: upper and lower limbs, trunk, gluteus muscles<br>Frequency: NR<br>Volume: 2-4sets x 8-12rep<br>Intensity: NR<br><br>Modality: balance and coordination<br>Type: one-legged stance, static heel/toes<br>Frequency: NR<br>Volume: NR<br>Intensity: N/A<br><br><u>Total (for all modalities):</u><br>Frequency: 2x/day<br>Volume: 30min	need (Nasal cannula, oxygen mask, Venturi mask, Non-rebreather mask), FiO_2, PaO_2 and SaO_2 (blood samples), CT pulmonary scan<br><br><u>Quality of life, mental health, and general state:</u><br>Disability level (BI), Ambulation capacity (method NR)<br><br><u>Biological parameters:</u><br>Arterial lactate, CRP, D-dimer, LDH
Daynes et al., 2021 [41]	Prospective Single group	COV+:<br>n=30 (14f, 16m); 58±16 yo; NR<br>Asthma (n=3), COPD (n=1), CVD (n=1)	**Moderate-to-High**<br>Hospital (n=26): 10±14 days<br>ICU and mechanical ventilation (n=5)	Duration between COVID-19 diagnosis and RP: 125±54 days	In hospital (center of exercise and rehabilitation sciences)<br><br>Physical activity, education<br><br>Duration: 6 wk	Modality: aerobic<br>Type: walking (without and with treadmill), cycling<br>Frequency: NR<br>Volume: NR<br>Intensity: NR<br><br>Modality: resistance<br>Type: lower and upper limbs<br>Frequency: NR<br>Volume: NR<br>Intensity: NR<br><br><u>Total (for all modalities):</u><br>Frequency: 2x/wk<br>Intensity: NR but used of dyspnea and RPE (Borg scale)	<u>Functional capacity:</u><br>Incremental shuttle walking test, ESWT<br><br><u>Respiratory function:</u><br>Respiratory symptoms (COPD Assessment Test)<br><br><u>Quality of life, mental health, and general state:</u><br>Fatigue (Functional Assessment of Chronic Illness Therapy - Fatigue score), Anxiety and depression (HADS), QoL (ED5D), Cognition (MoCA)
Dun et al., 2021 [42]	Retrospective Controlled trial, two groups	COV+ (≥5 performed RP sessions):<br>n=27 (18f, 9m); 54±16 yo; 23.8±2.5 kg/m²<br>CVD (n=6), DM (n=4), HT (n=3), Dyslipidemia (n=2), Smoking history (n=2), Cerebrovascular disease (n=2), Peptic ulcer (n=0), Cancer (n=0)<br><br>CON (COV+, <5 performed RP sessions):<br>n=71 (35f, 36m); 44±13 yo; 23.8±3.2 kg/m²<br>Smoking history (n=8), HT (n=8), Dyslipidemia (n=6), CVD (n=6), Cancer (n=4), DM (n=3), Peptic ulcer (n=3), Cerebrovascular disease (n=1)	**Moderate-to-High**<br>Hospitalization:<br>- COV+: 18±9 days<br>- CON: 17±8 days<br><br>Clinical classification of COVID-19:<br>Mild (COV+; n=0, CON: n=1), Moderate (COV+: n=17; CON: n=56), Severe (COV+: n=8; CON: n=10), Critical (COV+: n=2; CON: n=4)	2 wk after discharge from acute care	In hospital (rehabilitation center) and telerehabilitation (no-supervised)<br><br>Physical activity, respiratory exercises<br><br>Duration: 12 wk	<u>Supervised at hospital</u><br>Modality: aerobic<br>Type: cycling, walking (HIIT on ergocycle and treadmill)<br>Frequency: 3x/wk<br>Volume: 2sets x 4min/4min<br>Intensity: 4min high intensity (RPE=15-17, Borg scale 16-20)/4min low intensity (RPE<14)<br><br><u>Telerehabilitation</u><br>Modality: aerobic<br>Type: walking, jogging, cycling<br>Frequency: 2-5x/wk<br>Volume: 30-50min<br>Intensity: 40-60%HR_{max} (predicted: 220 - age), RPE=13-14 [Evolution after 1 mo: 40-80%HR_{max}, RPE=13-16]	*Post-RP tests were performed 10 wk after the end of the RP.*<br><br><u>Functional capacity:</u><br>6MWT<br><br><u>Biological parameters:</u><br>SARS-CoV-2 IgG and IgM, Lymphocytes, WBC, Neutrophils, Eosinophils, Hemoglobin, Platelets, Alanine aminotransferase, Aspartate aminotransferase, Bilirubin, Total protein, ALB, Globulin, Blood urea nitrogen, Urid acid, Creatinine (blood samples)

		Groups comparison:<br>age ($p<0.01$)					
Everaerts et al., 2021 [43]	Prospective<br>Single group	COV+:<br>n=22 (7f, 15m);<br>median: 54.5 [IQR: 47, 61] yo; median: 28 [IQR: 25, 31] kg/m²<br>DM (n=4, but 3 of them stopped anti-DM treatment during the program), CKD (n=5)<br><br>Previous history of rehabilitation: n=5 (5 to 80 days before RP for the study)	**Moderate-to-High**<br>Hospitalization: median: 29 [IQR: 12, 39] days<br>Mechanical ventilation (n=7): median: 14 [IQR: 11, 24] days<br>Among ICU patients (n=15): High-flow oxygen nasal cannula (n=1), Mechanically ventilated (n=14; among them n=1 Extracorporeal membrane oxygenation, n=2 Tracheostomy)<br><br>Need for oxygen at RP admission (n=2)	Duration between hospital discharge and RP: median: 47 [IQR: 15, 69] days	In hospital (outpatient)<br><br>Physical activity<br><br>Duration: 12 wk	Modality: aerobic<br>Type: walking, cycling, stepping (continuous or IT, on treadmill, ergocycle, arm ergometer, stair climbing, step)<br>Frequency: NR<br>Volume: NR<br>Intensity: started at 60-75%WR_{max} of CPET<br>[Evolution: Patients went from IT (for patients not able to cycle ≥ 10min on 80%WR_{max} during CPET) to continuous training before wk 6]<br><br>Modality: resistance<br>Type: lower and upper limbs (leg, chest press)<br>Frequency: NR<br>Volume: NR<br>Intensity: NR<br><br><u>Total (for all modalities):</u><br>Frequency: 3x/wk<br>Volume: 90min<br>Intensity: Dyspnea and RPE=4-6 (Borg scale 0-10)	<u>Functional capacity:</u><br>6MWT, CPET, Handgrip, Quadriceps strength (method NR)<br><br><u>Respiratory function:</u><br>FVC, FEV, TLC, DLCO, PI_{max}, PE_{max} (methods NR)<br><br><u>Quality of life, mental health, and general state:</u><br>Anxiety and depression (HADS), Cognitive deficits (reported by patients), Cognition (MoCA)
Ferraro et al., 2021 [44]	Prospective<br>Case series	COV+:<br>n=7 (2f, 5m);<br>65.7±11.9 yo; NR<br>HT (n=2), DM2 (n=1), Liver disease (n=1), CKD (n=1), Smoking history (n=1)	**Moderate-to-High**<br>Hospitalization: 27±11 days (calculated from raw data)<br>ICU (n=2): 15 and 18 days<br><br>COVID-19 complications: Bilateral pneumonia (n=7), Peripheral neuropathy (n=3), Pulmonary embolism (n=1), Stroke (n=1)	NR	In hospital (COVID-19 rehabilitation unit)<br><br>Physical activity, respiratory exercises, kinesitherapy<br><br>Duration: NR	Modality: aerobic<br>Type: walking [for progressive distances]<br>Frequency: NR<br>Volume: NR<br>Intensity: NR<br><br>Modality: resistance<br>Type: lower and upper limbs, trunk, gluteus muscles<br>Frequency: NR<br>Volume: NR<br>Intensity: NR<br><br>Modality: balance and coordination<br>Type: one-legged stance, static heel/toes<br>Frequency: NR<br>Volume: NR<br>Intensity: NR<br><br><u>Total (for all modalities)</u>:<br>Frequency: 6x/wk, 1-2x/day<br>Volume: 30min	<u>Functional capacity:</u><br>6MWT, Handgrip, 10MWT, SPPB (score 0-12 from 3 tests: static body balance, ability to walk, and stand up from a chair)<br><br><u>Quality of life, mental health, and general state:</u><br>Fatigue (Borg scale 0-10), Disability level (FIM, BI)
Gloeckl et al., 2021 [45]	Prospective<br>Two groups	M.COV+ (mild/moderate COV+):<br>n=24 (20f, 4m);<br>median: 52 [IQR: 47, 56] yo; median: 24.7 [IQR: 22.0, 29.8] kg/m²<br>Obstructive sleep apnea (n=9), Chronic lung disease (n=7),	**Low-to-High**<br><br>M.COV+: **Low** (no hospitalization)<br><br>S.COV+: **Moderate-to-High**<br>Hospitalization: 26 days<br>ICU (n=22): median: 28 [IQR: 15, 40] days<br>Need for oxygen (n=24)<br>Mechanical ventilation (n=15): median: 18 [IQR: 11, 43] days	NR for M.COV+<br><br>S.COV+: median: 18 days [IQR: 5, 40] after hospital discharge	In hospital<br><br>Physical activity, education, respiratory exercises, activities of daily living training, relaxation techniques, occupational therapy, psychological support, nutritional counselling, drug therapy	Modality: aerobic<br>Type: cycling<br>Frequency: 5x/wk<br>Volume: 10-20min<br>Intensity: 60-70%WR_{peak} (NR if intensity was estimated or defined from a previously test)<br><br>Modality: resistance<br>Type: machines (leg press, knee extension, pull-down, push-down, and	<u>Functional capacity:</u><br>6MWT<br>Only for S.COV+: ESWT, HR pre- and post-ESWT, Maximal isometric knee extension, Handgrip, 5-rep STST<br><br><u>Respiratory function:</u><br>FVC, FEV1, TLC, DLCO, KCO (plethysmography), PaO_2 and $PaCO_2$ (blood samples), Dyspnea post-6MWT (Borg scale, probably 0-10), Need for oxygen therapy during rest and exertion<br>Only for S.COV+: SaO_2 and breathing frequency pre- and post-ESWT, Dyspnea during daily activities (MRC Dyspnea scale)

		Smoking history (n=7), Obesity (n=5), HT (n=5), Dyslipidemia (n=3), Coronary heart disease (n=1), DM (n=1), CKD (n=0), Stroke (n=0)<br>Comorbidities/patient: median: 2 [IQR: 2, 4]<br><br>S.COV+ (severe/critical COV+):<br>n=26 (8f, 18m); median: 66 [IQR: 60, 71] yo; median: 26.9 [IQR: 24.2, 29.2] kg/m²<br>Smoking history (n=20), HT (n=16), Dyslipidemia (n=10), Obstructive sleep apnea (n=9), Coronary heart disease (n=7), DM (n=6), CKD (n=6), Chronic lung disease (n=5), Obesity (n=5), Stroke (n=1)<br>Comorbidities/patient: median: 3 [IQR: 3, 5]			Duration: 3 wk	if possible: butterfly, forward/backward, rowing, back extension, abdominal trainer)<br>Frequency: 5x/wk<br>Volume: 3sets x 15-20rep (30min)<br>Intensity: NR	<u>Quality of life, mental health, and general state:</u><br>QoL (SF-36)<br>Only for S.COV+: Anxiety (GAD-7), Depression (PHQ-9), Cognition (MoCA), COVID-19 symptoms<br><br><u>Biological parameters:</u><br>CRP, Leukocytes, D-dimer, Pro-BNP
Gobbi et al., 2021 [46]	Prospective<br>Two groups	Total group:<br>n=34 (18f, 16m); NR; NR<br>HT (n=6), CVD (n=6), DM (n=3)[µ]<br><br>S.COV+ (COV+ with sarcopenia):<br>n=20 (9f, 11m); 71.5±17.0 yo; 21.0±4.2 kg/m²<br>Comorbidities NR<br><br>NS.COV+ (COV+ without sarcopenia):<br>n=14 (9f, 5m); 68.0±16.5 yo; 27.3±9.0 kg/m²<br>Comorbidities NR<br><br>Groups comparison: age (p-value NR), BMI (p-value NR)	**Moderate-to-High**<br>ICU (Total group: n=11; S.COV+: n=8; NS.COV+: n=3)	Probably immediately after hospital discharge	In hospital (rehabilitation unit from COVID hospital)<br><br>Physical activity, psychiatric support, congitive behavioral therapy, nutritional treatment<br><br>Duration: 28 days	Modality: aerobic<br>Type: cycling (ergocycle), arm ergometer<br>Frequency: NR<br>Volume: NR<br>Intensity: 65%HR_{max} (predicted: (220-age)x0.65)<br><br>Modality: resistance<br>Type: sitting-to-bed exercises for upper-body conditioning and progressive limb muscle strengthening<br>Frequency: NR<br>Volume: 1-3sets x 8-12rep (2min recovery between sets)<br>Intensity: NR<br><br><u>Total (for all modalities):</u><br>Frequency: 6x/wk<br>Volume: 45min<br>Intensity: Individualized with patient's conditioning, subjective perception of fatigue and clinical status. RPE (Borg scale 0-10) and SaO_2 was monitored during exercise.	<u>Functional capacity:</u><br>Handgrip, Time-up-to-go test (>13.5s = practical cut-off for high fall risk, >20s = severe sarcopenia)<br><br><u>Respiratory function:</u><br>Dyspnea (Borg scale 0-10) and SaO_2 in lying, sitting and standing positions, and at the end of Time-up-to-go test<br><br><u>Biological parameters:</u><br>ALB, Ferritin, Vitamin D<br><br><u>Other:</u><br>BMI, Phase angle and ASM (obtained from impedancemetry with the whole-body resistance and the reactance, ASM<20 for m and ASM<15 for f = sarcopenia)
Güler et al., 2021 [47]	Retrospective<br>Single group	COV+:<br>n=85 (22f, 63m); 58.3±11.1 yo; 25.3±4.8 kg/m² (range: 17.7-46.7)<br>HT (n=42), DM	**Moderate-to-High**<br>Hospitalization: 16±12 days (range: 1-60)<br>ICU (n=67): 19±19 days (range: 0-120)<br><br>Respiratory status (probably at	Immediately after post-acute care	In hospital (COVID-19 rehabilitation clinic)<br><br>Physical activity (Neuromuscular rehabilitation, n=77;	<u>"Neuromuscular rehabilitation"</u><br>Modality: aerobic<br>Type: walking (in the parallel bar and the hall), step climbing<br>Frequency: NR	<u>Respiratory function:</u><br>Need for oxygen therapy<br><br><u>Quality of life, mental health, and general state:</u><br>Disability level (BI), Functional status

		(n=29), Smoking history (n=13), COPD (n=14), CVD (n=14), Thyroid disease (n=10), Rheumatic disorders (n=5), CKD (n=1), Cancer (n=1)	admission to RP): Nasal oxygen cannula (n=77), Reservoir mask (n=53), High flow oxygen therapy (n=33), Non-invasive ventilator (n=5), Mechanical ventilation (n=31)<br><br>COVID-19 complications: Pulmonary involvement (n=48), Guillain-Barré syndrome (n=18), Critical illness-related myopathy/neuropathy (n=11), Acute cerebrovascular disease (n=8), Dysphagia (n=5), Steroid myopathy (n=1), Sinus vein thrombosis (n=1)		Arm/cycle ergometer, n=44), electrostimulation (Without cycling, n=43; With cycling, n=29), respiratory exercises (High-frequency chest oscillation devices: n=10), speech and swallowing therapy (n=5), nutritional and psychological support<br><br>Duration: at least 3 wk for Arm/cycle ergometer program (no further information)	Volume: NR<br>Intensity: NR<br><br>Modality: resistance<br>Type: active and passive exercises<br>Frequency: NR<br>Volume: NR<br>Intensity: NR<br><br>Modality: balance and coordination<br>Type: standing exercises<br>Frequency: NR<br>Volume: NR<br>Intensity: NR<br><br><u>"Arm/cycle ergometer"</u><br>Modality: aerobic<br>Type: "arm cycle" ergometer<br>Frequency: 5x/wk<br>Volume: 20-30min<br>Intensity: 40-60%WR_{max} (NR if intensity was estimated or defined from a previously test)	
Hameed et al., 2021 [48]	Prospective Controlled trial, three interventional groups and one control group	VPT COV+ ('Virtual physical therapy')[µ]: n=44 (25f, 19m); median: 60 [IQR: 14][µ] yo; median: 30.6 [IQR: 8.2][µ] kg/m²<br>(n=27)<br>HT (n=22), DM (n=11), Other pulmonary diseases (n=8), CVD (n=6), Autoimmune disorder (n=5), Asthma (n=4)<br><br>HPT COV+ ('Home in-person physical therapy')[µ]: n=25 (6f, 19m); median: 57 [IQR: 14][µ] yo; median: 28.4 [IQR: 5.2][µ] kg/m²<br>(n=24)<br>HT (n=15), DM (n=13), CVD (n=4), Other pulmonary diseases (n=4), Asthma (n=3), Autoimmune disorder (n=2)<br><br>EI COV+ ('Independent exercise')[µ]: n=17 (6f, 11m); median: 59 [IQR: 20][µ] yo; median: 29.2 [IQR: 6.7][µ] kg/m²<br>(n=13)<br>HT (n=11), DM (n=6), CVD (n=2), Asthma (n=1),	**Moderate-to-high**<br>Hospitalization:<br>- VPT: median: 14 [IQR: 14][µ] days<br>- HPT: median: 39 [IQR: 30][µ] days<br>- EI: median: 9 [IQR: 17][µ] days<br>- CON: median: 13 [IQR: 33][µ] days<br>Intubation:<br>- VPT (n=6): median: 13 [IQR:11][µ] days<br>- HPT (n=16): median: 20 [IQR: 15][µ] days<br>- EI (n=5): median: 10 [IQR: 15][µ] days<br>- CON (n=5): median: 13 [IQR: 17][µ] days<br><br>Duration of hospitalization effect between groups (p<0.001)	Unclear	Telerehabilitation (supervised sessions and independently sessions)<br><br>Physical activity, respiratory exercises<br><br>Duration: 4-6 wk	Modality: resistance<br>Type: circuit training detailed in a guide for the patients (sit to stand, standing walk, shoulder caption, standing heel raises, sidestepping, wall pushups) or detailed in videos on Internet (for phase 4)<br>Frequency: 1set 4x/day everydays (phase 1); 2sets 2x/day, 6x/wk (phase 2); 3sets 2x/day, 6x/wk (phase 3); 1-2 sessions/wk were tele-supervised<br>Volume: 1-3sets; alternance of 30s work/30s rest (30-60min) [Evolution: increase until 1min work/1min rest (with stable vitals at rest: SaO_2>90%, RPE<3 and 'HR within acceptable activity-specific increases with exercise') and then pass in the next phase]<br>Intensity: NR, but HR, SaO_2 (always>88%) and RPE (Borg scale 0-10) were monitored during exercise. If more rest was needed, patients were able to rest until they achieved RPE≤3 or SaO_2>90%.	<u>Functional capacity:</u><br>2-min step test, 30-s STST<br><br><u>Respiratory function:</u><br>SaO_2 (probably at rest)

		Autoimmune disorder (n=1), Other pulmonary diseases (n=0)<br><br>CON (COV+ whitout intervention):<br>n=20 (11f, 9m); median: 58 [IQR: 18]$^{\mu}$ yo; median: 28.7 [IQR: 9.7]$^{\mu}$ kg/m² (n=13)<br>HT (n=11), DM (n=8), CVD (n=3), Other pulmonary diseases (n=2), Asthma (n=0), Autoimmune disorder (n=0)<br><br>Groups comparison: gender effect between groups (p<0.05)					
Hayden et al., 2021 [49]	Prospective<br>Three groups	Total groups (COV+): n=108 (49f, 59m); 55.6±10.1 yo (range: 32-85); 30.2±6.1 kg/m² (range: 19.3-53.3)<br>CVD (n=60), Obesity (n=46), Orthopedic comorbidities (n=44), Upper or lower airway disease (n=34, among them, COPD n=2 and Asthma n=18), Hyperlipoproteinemia (n=28), Psychological comorbidities (n=27), Neurological comorbidities (n=24), DM (n=15), No comorbidity (n=2)<br><br>Group A (severe COV+ with hospital discharge < 1 mo): n=55 (21f, 34m); 57.9±10.8 yo (range: 33-85); 29.9±5.7 kg/m² (range: 19.3-53.3)<br>Comorbities NR<br><br>Group B (severe COV+ with hospital discharge > 1 mo): n=32 (11f, 21m); 54.0±9.9 yo (range: 32-80); 31.5±6.7 kg/m² (range: 21.0-48.3)	**Moderate-to-High**<br>Hospitalization:<br>- Total groups: 26±18 days (range: 1-101)<br>- Group A: 32±19 days (range: 3-101)<br>- Group B: 19±11 days (range: 3-49)<br>- Group C (n=5): 1 night maximum<br>ICU:<br>- Total groups (n=57): 24±17 days (range: 5-97)<br>- Group A (n=39): 22±18 days (range: 4-97)<br>- Group B (n=18): 15±9 days (range: 5-40)<br>- Group C: N/A<br>Invasive ventilation:<br>- Total groups (n=39): 18±16 days (range: 5-87)<br>- Group A (n=27): 20±18 days (range: 8-87)<br>- Group B (n=12): 12±5 days (range: 5-23)<br>- Group C: N/A<br>Need for oxygen therapy (% of sample):<br>- Total groups: 68%<br>- Group A: 87%<br>- Group B: 69%<br>- Group C: 14%<br>Groups comparison: Group C < Groups A and B<br><br>Most important symptoms (% of total sample): Dyspnea (50%), Anxiety and worries regarding one's own health status (17%), Faintness/lack of energy (16%), Pain in various parts of the body (8%)	Duration between the acute phase or hospital discharge and RP:<br>- Total groups: 69±75 days (range: 0-270)<br>- Group A: 11±11 days (range: 0-31) (n=24 immediately after hospital discharge)<br>- Group B: 121±70 days (range: 32-270)<br>- Group C: 143±55 days (range: 35-270)	In hospital (inpatient rehabilitation center)<br><br>Physical activity and vibration training if necessary, respiratory exercises, patient information about COVID-19, routine medical diagnosis, psychological support, and if necessary, daily activities life training, nutritional counseling, occupaional therapy<br><br>Duration (Total group): 26±6 days (range: 5-42 days)	Modality: aerobic<br>Type: cycling (probably on ergocycle), nordic walking, indoor sports<br>Frequency: 3-5x/wk<br>Volume: 30-60min<br>Intensity: for cycling, based on 6MWT performance (RPE=4-6, Borg scale 0-10)<br><br>Modality: resistance<br>Type: machines (leg press, rowing pull, latissimus pull, butterfly reverse, cable pull, abdominal exercises)<br>Frequency: 2-3x/wk<br>Volume: 3sets x 12rep<br>Intensity: 12RM<br><br>Modality: vibration (if necessary)<br>Type: statically in squat position<br>Frequency: 7x/wk<br>Volume: 3sets x 1-2min<br>Intensity: 16-26 Hz (1.5-4 amplitude) (the goal was muscular exhaustion at the end of the set and the session)	Functional capacity:<br>6MWT<br><br>Respiratory function:<br>VC, TLC, FEV1, DLCO, PI_{max}, RV, total specific airway resistance (spirometry and plethysmography), PaO_2 and $PaCO_2$ (blood samples), Dyspnea at rest and exertion (11-point NRS, MRC Dyspnea scale)<br><br>Quality of life, mental health, and general state:<br>QoL (EQ5D 5 levels, EQ5D 5 levels VAS), Fatigue (Brief Fatigue Inventory), Depression (PHQ-9), Anxiety (GAD-7), Cough, Sputum and pain (11-point NRS), Global rating of change in subjective health<br><br>Biological parameters:<br>D-dimer, CRP, BNP, LDH<br><br>Adherence and tolerability:<br>Estimation of the rehabilitation effectivness (Likert scale 0-10)

		Comorbities NR<br><br>Group C (mild COV+):<br>n=21 (17f, 4m);<br>52.1±6.8 yo (range: 39-61); 28.9±6.1 kg/m² (range: 19.9-46.0)<br>Comorbities NR<br><br>Groups comparison: significant differences between Group C with Groups A and B for gender repartition (p<0.001) and age (p<0.01)					
Hermann et al., 2020 [50]	Retrospective<br>Two groups	Total groups (COV+):<br>n=28 (14f, 14m);<br>66.0±9.3 yo; 27.6±4.9 kg/m²<br>HT (n=14), DM2 (n=7), Smoking history (n=7), Dyslipidemia (n=6), COPD (n=6), CKD (n=5), Coronary artery disease (n=4), Peripheral artery disease (n=2), Stroke (n=1)<br><br>Ventilation+ (COV+):<br>n=12 (3f, 9m);<br>64.3±8.9 yo; 26.9±3.5 kg/m²<br>HT (n=5), Smoking history (n=5), DM2 (n=4), CKD (n=3), Dyslipidemia (n=2), Coronary artery disease (n=1)<br><br>Ventilation- (COV+):<br>n=16 (11f, 5m);<br>67.4±9.7 yo; 28.1±5.7 kg/m²<br>HT (n=9), COPD (n=6), Dyslipidemia (n=4), Coronary artery disease (n=3), DM2 (n=3), Peripheral artery disease (n=2), CKD (n=2), Smoking history (n=2), Stroke (n=1)	**Moderate-to-High**<br>Hospitalization:<br>- Total groups: 19.3±10.7 days<br>- Ventilation+: 27.6±9.1 days<br>- Ventilation-: 12.7±6.5 days<br>ICU:<br>- Total groups (n=17): 13.9±7.3 days<br>- Ventilation+ (n=12): 17.0±5.9 days<br>- Ventilation- (n=5): 6.4±3.8 days<br><br>Respiratory status at RP admission (Total groups): Need for non-invasive ventilation (n=3, only during night time), Need for oxygen therapy (n=21)<br><br>85% of positive patients for COVID-19 at admission<br><br>Groups comparison: hospitalization and ICU duration (p<0.001)	Duration of hospitalization before admission to RP (Total groups):<br>19.3±10.7 days<br><br>(Initially admitted in the RP after being asymptomatic for 2 days and 10 days after onset of infection, and later admitted if at least one negative swab before transfer)	In hospital<br><br>Physical activity, respiratory exercises, education, nutritional/diabetes advices, structured smoking cessation program if needed, psychosocial support, massages<br><br>Duration: 2-4 wk (20 days)	Modality: aerobic<br>Type: walking (in- or out-door), cycling (ergocycle)<br>Frequency: NR<br>Volume: NR<br>Intensity: NR (but derived from initial 6MWT. Criteria for stopping or reducing exercise intensity was SaO$_2$<88%, symptom limited (RPE≥6) or/and reaching submaximal HR). [Evolution: adjusted continuously to achieve the maximum tolerated exercise load[µ]]<br><br>Modality: resistance<br>Type: NR<br>Frequency: NR<br>Volume: 3sets x 20rep<br>Intensity: 20RM[µ]<br><br><u>For isolated patients:</u><br>Frequency: 1-2x/day<br>Volume: 15-45min<br>Intensity: limited<br>Instructions for self-training<br><br><u>Total (for all RP):</u><br>Frequency: 5-6x/wk<br>When a drop in SaO$_2$ was observed, oxygen was added with a maximum of 4L *via* nasal cannula to keep SaO$_2$>90%	<u>Functional capacity:</u><br>6MWT<br><br><u>Respiratory function:</u><br>SaO$_2$ (probably at rest), Need for oxygen therapy<br><br><u>Quality of life, mental health, and general state:</u><br>Patients' feeling (Feeling Thermometer)
Imamura et al., 2021 [51]	Retrospective<br>Single group	COV+:<br>n=27 (7f, 20m);<br>53.8±13.3 yo;<br>27.6±5.8 kg/m²<br>For n=23 (4 'drop-out'): HT (n=12), DM	**High** (n=23)<br>Hospitalization: 75±40 days (range: 20-164)<br>ICU: 30±18 days (range: 5-76)<br>Intubation: 23±14 days	Immediatly after hospital discharge	In hospital (inpatient rehabilitation institute)<br><br>Physical activity, electrostimulation, speech, psychology, nutrition and	Modality: aerobic<br>Type: cycling (ergocycle)<br>Frequency: NR<br>Volume: NR<br>Intensity: NR	<u>Functional capacity:</u><br>Muscle strength (MRC score 1-60), SPPB (score 0-12 from 3 tests: static body balance, ability to walk and stand up from a chair)<br><br><u>Respiratory function:</u><br>PI$_{max}$ and PE$_{max}$ (manovacuometer)

		(n=10), Smoking history (n=6), Obesity (n=3), Arrhythmia (n=2), CVD (n=2), Liver disease (n=1), Psychiatric illness (n=1), Dyslipidemia (n=1)			dietetic, nursing, social services, and cognitive therapies, robot-assisted and virutal reality-assisted rehabilitation according to individual prescriptions<br><br>Duration: 23±9 days	Modality: resistance<br>Type: NR<br>Frequency: NR<br>Volume: NR<br>Intensity: NR<br><br>Modality: balance<br>Type: balance, gait, and body awareness training<br>Frequency: NR<br>Volume: NR<br>Intensity: NR<br><br>Total (for all modalities):<br>Frequency: 2-3x/wk for resistance and aerobic training<br>Intensity: NR but RPE (Borg scale NR) was monitored	Quality of life, mental health, and general state:<br>Ambulation capacity (FAC), Disability level (FIM)<br><br>Other:<br>FFM (bioimpedance, skinfolds and circumferences), Body weight, BMI, Functional Oral Intake Scale, Dysphagia (oropharyngeal dysphagia classification), Malnutrition (categories of the Global Leadership Initiative for Malnutrition)
Li et al., 2021 [52]	Retrospective Single group	COV+:<br>n=16 (8f, 8m);<br>70.1±16.5 yo;<br>22.6±3.3 kg/m² (calculated from raw data)<br>CKD (n=10), HT (n=8), CVD (n=4), DM2 (n=4), Emphysema/COPD (n=4), Ischemic stroke (n=3), fatty liver (n=2), Alzheimer (n=2), Anemia (n=2), Hypercholesterolemia (n=1), Bile stone (n=1), Chronic renal ischemia (n=1), Hypoproteina (n=1), Breast cancer (n=1), Prostate surgery (n=1), Low toxine levels (n=1), Dysphagia (n=1)	**High**<br>Hospitalization: 27.7±8.7 days (range: 11-46) (calculated from raw data)<br>ICU: 17.4±9.7 days (range: 6-38) (calculated from raw data)<br><br>Respiratoy status at RP admission:<br>- Mechanical ventilation (n=3): 17.3±11.5 days (calculated from raw data)<br>- Breathing spontaneously with high-flow nasal oxygenation or nasal cannulae (n=13)	Duration between admission to hospital and the beginning of RP[µ]: 8.9±7.7 days (calculated from raw data)<br>In the middle of the disease (during ICU)	In hospital<br><br>Physical activity, respiratory exercises, speech therapy, drug therapy<br><br>Program of physiotherapy inspired from Gosselink et al. 2011<br><br>Duration: 10.6±7.1 days	Modality: N/A<br>Type: rolling over and moving on the bed regularly, sitting up in bed, sitting on the bedside, sitting on a chair, standing, walking (along a 7m walkway)<br>Frequency: NR<br>Volume: NR<br>Intensity: SaO_2>90%, Dyspnea<4 (Borg scale 0-10). [Evolution: walking program increase by 10m each day, with 2-3 short rest periods during walking]<br><br>Total (for all RP):<br>Frequency: 2x/day<br>Volume: 30-40min<br><br>For ventilated patients (n=3): only passive/assisted active limb mobility	Function capacity:<br>Walking distance managed by patients independently or with the help of an assistant (not described as a 6MWT), Muscle strength (MRC score 1-60), Physical function (Physical Function in Intensive Care Test), Mobility (De Morton Mobility Index)<br><br>Respiratory function:<br>PaO_2 and FiO_2 (method NR), PEF (method NR), PI_{max} (respiratory pressure meter), Dyspnea at rest (Borg Scale, probably 0-10)<br><br>Quality of life, mental health, and general state:<br>Disability level (BI)
Martin et al., 2021 [53]	Prospective Controlled trial, two groups	COV+:<br>n=14 (3f, 14m);<br>60.8±10.4 yo (range: 55.6-65.9); 29.7±5.2 kg/m² (range: 27.3-32.2)<br>Comorbidities NR<br><br>CON (COV+ subjects refusing the RP):<br>n=13 (7f, 6m);<br>61.9±10.7 yo (range: 57.9-66.0); 28.1±3.9 kg/m² (range: 26.0-30.3)<br>Comorbities NR	**High**<br>Hospitalization:<br>- COV+: 16±10 days<br>- CON: 16±9 days<br>ICU (COV+: n=4; CON: n=2)<br><br>Need for oxygen at RP admission (COV+: n=12; CON: n=12)	Immediately after hospital discharge	Telerehabilitation (supervised and no-supervised)<br><br>Physical activity<br><br>Duration: 5 wk	Modality: aerobic<br>Type: NR<br>Frequency: 2x/wk<br>Volume: 30min<br>Intensity: RPE=6 (Borg scale, probably 0-10)<br><br>Modality: resistance<br>Type: upper and lower limbs (with bottle of water and a chair)<br>Frequency: 2x/wk<br>Volume: 2-3sets x 8-12rep (20min)<br>Intensity: NR<br><br>Patients were then encouraged to perform undersupervised exercises 3x/wk using provided templates.	*Post-RP test were performed 3 mo after the beginning of RP.*<br><br>Functional capacity:<br>1-min STST, HR pre- and post-STST<br><br>Respiratory function:<br>SaO_2 and Dyspnea pre- and post-STST (VAS 1-10)
Mohamed et al., 2021 [54]	Prospective Randomized controlled	COV+:<br>n=15 (7f, 8m);<br>44.6±4.3 yo; 24.7±1.3 kg/m²	**Moderate**<br>Hospitalization[µ]<br>Patients with mild or moderate COVID-19 (no or low-grade fever)	Unclear, maybe during/after the acute phase	Telerehabiltation (no-supervised)	Modality: aerobic<br>Type: walking/running or cycling (MICT on treadmill and ergocycle)<br>Frequency: 3x/wk	Quality of life, mental health, and general state:<br>QoL (Wisconcin Upper Respiratory Symptom Survey-24)<br><br>Biological parameters:

	trial, two groups	Smoking history (n=4) - No comorbidity<br><br>CON (COV+ without RP):<br>n=15 (8f, 7m); 35.3±4.0 yo; 24.0±1.2 kg/m²<br>Smoking history (n=3) - No comorbidity<br><br>Patients with other chronic diseases (such as heart problems, HT, or DT), and women with contraception were excluded.			Physical activity, drug therapy<br><br>Duration: 2 wk for physical activity and 5 days for drug therapy	Volume: 40min<br>Intensity: 5min of warm-up + 30min 60-75%HR_{max} (predicted: 210-age), RPE=12-14 (Borg scale 6-20) + 5min of cool-down	Lymphocytes, Leukocytes, IL-6, IL-10, TNF-α (blood samples), IgA-S concentration (salivary samples)
Ozyemisci Taskiran et al., 2021 [55]	Prospective Controlled trial, two groups	COV+:<br>n=18 (7f, 11m); median: 73 yo (range: 55-91); median: 24.8 kg/m² (range: 16.0-39.1)<br>Chronic pulmonary disease (n=8, and among them: Interstitial lung disease (n=3), Cancer (n=2), COPD (n=2), Tuberculosis (n=1)), HT (n=7), DM (n=6), Cancer (n=6), Coronary artery disease (n=5), Neurologic disease (n=4)<br><br>CON (COV+ without RP):<br>n=17 (4f, 13m); median: 70 yo (range: 49-88); median: 29.4 kg/m² (range: 22.8-39.7)<br>HT (n=10), DM (n=6), Coronary artery disease (n=5), Cancer (n=2), COPD (n=1)	**High**<br>Hospitalization:<br>- COV+: median: 27 days (range: 6-52)<br>- CON: median: 16 days (range: 8-26)<br>ICU:<br>- COV+: median: 16 days (range: 2-30)<br>- CON: median: 9 days (range: 1-20)<br>Stay in ICU≤5 days (COV+: n=5; CON: n=6)<br>Invasive mechanical ventilation:<br>- COV+ (n=13): median: 17 days (range: 5-27)<br>- CON (n=13): median: 7 days (range: 1-19)<br><br>COVID-19 complications: Pressure ulcers (COV+: n=13; CON: n=8), Co-infection (COV+: n=12; CON: n=8), Sepsis (COV+: n=10; CON: n=11)	During ICU (≥5 days of the ICU stays, ≥10 days after the onset of symptoms)	In hospital (ICU)<br><br>Physical activity, drug therapy<br><br>Number of sessions: median 6 (range: 2-14)<br><br>Effective program in n=11 patients (who fulfilled the inclusion criteria and ICU stay>5 days)	For invasively mechanically ventilated and sedated patients:<br>Modality: N/A<br>Type: passive range of motion exercises<br>Frequency: 6x/wk<br>Volume: 10-15rep (15min)<br>Intensity: NR<br><br>For non-invasively mechanically ventilated patients:<br>Modality: N/A<br>Type: active-assistive or active range of motion exercises in-bed according to their condition, including sitting unspported, sit to stand and walking (commenced following their transfer to the ward)<br>Frequency: NR<br>Volume: NR<br>Intensity: NR	Functional capacity:<br>Handgrip, Muscle strength (MRC score 1-60)<br><br>Quality of life, mental health, and general state:<br>Physical function, Pain, Role limitations-physical, Role limitations-emotional, Mental health, Tremor in the hands, Social functioning, Energy, QoL (SF-36)
Pancera et al., 2021 [56]	Retrospective Case series	COV+:<br>n=7 (7m); 48.3±8.7 yo (range: 37-61); 24.7±3.7 kg/m² (calculated from raw data)<br>Smoking history (n=7), HT (n=4)	**High**<br>ICU: 18.4±2.8 days (calculated from raw data)<br>Tracheostomy: 8.4±3.6 days (calculated from raw data)<br><br>Patients still positived for COVID-19, requiring mechanical ventilation (n=6) and isolated in a COVID unit at RP admission (T0)	During the acute phase	In hospital (rehabilitation center)<br><br>Physical activity, electrostimulation, respiratory exercises<br><br>Duration: 2-5 wk<br><br>Calculated from raw data:<br>- T0 to T1: 11±5 sessions	Modality: aerobic (as soon as possible)<br>Type: walking (with or without ambulatory assisting), climbing and descending stairs (from T1), cycling (ergocycle) (from T1)<br>Frequency: NR<br>Volume: 30min for cycling<br>Intensity: 20W, RPE=4-6 (probably Borg scale 0-10) for cycling	*Tests were performed at pre-RP, at the end of T1, and at the end of T2 (post-RP).*<br><br>Functional capacity:<br>Muscle strength (MRC score 1-60), SPPB (score 0-12 from 3 tests: static body balance, ability to walk and stand up from a chair)<br><br>Rerspiratory function:<br>PI_{max} and PE_{max} (MicroRPM - Respiratory Pressure Meter - at T1 and T2 only)<br><br>Quality of life, mental health, and general state:

					(range: 6-20)<br>- T1 to T2: 9±4 sessions (range: 0-17)	[Evolution: increase of 10W when RPE and dyspnea<4]<br><br>Modality: resistance (as soon as possible)<br>Type: sit-to-stand, strength exercises with elastic bands or free weights (from T1)<br>Frequency: NR<br>Volume: 5-10rep for sit-to-stand, 2sets x 20rep for strength exercises<br>Intensity: NR for sit-to-stand, 50%1RM for strength exercises<br>[Evolution: 2sets x 10rep at 70%1RM for strength exercises]<br><br>Total (for all RP):<br>Frequency: 5x/wk<br>Volume: 45min	QoL (EDQ5 3 levels), Disability status (BI, BI based on dyspnea)<br><br>Other:<br>BMI, Quadriceps girth (10cm above the patella)
Patel et al., 2021 [57]	Retrospective Single group	COV+:<br>n=106 (27f, 79m); 64±14 yo; NR<br>Vascular or metabolic disease (n=43), History of cognitive disorder (n=6)	**High**<br>Hospitalization: 53±28 days<br>ICU (most of patients): 35±24 days<br>Intubation: median: 13 [IQR: 0, 20.5] days<br>Tracheostomy (n=33)	Probably immediately after acute hospital discharge	In hospital (COVID-19 recovery unit)<br><br>Physical activity, occupational therapy, and if necessary, speech-language therapy, neuropsychology, psychiatry, neurology, and pulmonology consultation, swallow therapy<br><br>Duration: 17±9 days	Modality: aerobic<br>Type: cycling (sitting ergocycle), walking<br>Frequency: NR<br>Volume: 10 steps at a time for walking<br>Intensity: NR<br><br>Modality: resistance<br>Type: upper and lower limbs ('TherEx with/without TheraBands", "TheraBands', "NutStep", free weight, weight bearing). Excentric movements are favoured while isometric and concentric movements are limited.<br>Frequency: NR<br>Volume: NR<br>Intensity: NR<br><br>Modality: balance<br>Type: standing reaching tasks, dribble soccer ball, shoot basketball<br>Frequency: NR<br>Volume: NR<br>Intensity: NR<br><br>Total (for all modalities):<br>Frequency: 2-3x/wk to 5-6x/wk<br>Volume: 30min to 60-90min<br>Intensity: rest breaks based on HR and SaO_2 of patients	Functional capacity:<br>Distance ambulated<br><br>Respiratory function:<br>Respiratory status<br><br>Quality of life, mental health, and general state:<br>QoL (AMPAC for basic mobility, AMPAC for daily activities), Ambulatory status
Piquet et al., 2021 [58]	Retrospective Single group	COV+:<br>n=100 (34f, 66m); median: 66 [IQR: 22]$^{\mu}$ yo; 26.0±5.4 kg/m²<br>HT (n=48$^{\mu}$), DM (n=29), Renal failure (n=13), Stroke (n=9), Immunosuppression (n=3), Coronapathy (n=1)	**Moderate-to-High**<br>No-ICU hospitalization (n=77): 11.7±6.5 days<br>Need for nasal oxygen$^{\mu}$ (n=77)<br>ICU (n=23): 13.8±9.0 days<br>Hospitalization after ICU (n=23): 10.2±4.9 days<br>Intubation (n=13): 8.2±8.5 days<br><br>Need for nasal oxygen at RP admission$^{\mu}$ (n=58)<br>Symptoms/complications due to COVID-19 at RP adimission: Dyspnea	Duration between onset of symptoms and admission in RP$^{\mu}$: 20.4±10.0 days (unclear if it is mean and SD or median and IQR)	In hospital (COVID-19 rehabilitation unit)<br><br>Physical activity, respiratory exercises, education, occupational therapy, speech therapy, psychology therapy<br><br>Duration: 9.8±5.1$^{\mu}$ days	Modality: aerobic<br>Type: cycling (ergocycle), 'pedal boards', arm ergometer<br>Frequency: NR<br>Volume: NR<br>Intensity: 'submaximal'<br><br>Modality: resistance<br>Type: sit-to-stand, tiptoe stand, squats (elastic bands, weights, dumbbells)<br>Frequency: NR<br>Volume: 3sets x 10rep for each exercise (depending on patients'	*Among included patients, functional outcome criteria were analyzed for lengths of stay ≥72h*<br><br>Functional capacity:<br>10-rep STST (time to perform 10 full sit-to-stands; when 10 STST could not be completed, the number of completions in 1min was collected), HR, and RPE (Borg scale, probably 0-10) pre and post-STST, Handgrip<br><br>Respiratory function:<br>Respiratory rate (method NR) and SaO_2 pre- and post-STST, Need for oxygen therapy

		Age>70 yo (n=41), BMI>30 (n=17)	(n=79), Asthenia (n=76), Fever (n=73), Cough (n=64), Myalgia (n=33), Diarrhea (n=25), Ageusia (n=16), Headache (n=14), Anosmia (n=13), Pulmonary embolism (n=4), Thrombosis (n=1)<br><br>At the time of the diagnosis: 26% had more than 50% pulmonary damage on CT scans.			abilities)<br>Intensity: NR<br><br>Total (for all modalities):<br>Frequency: 2x/day, 5days/wk<br>Volume: <20min	Quality of life, mental health, and general state:<br>Disability level (BI)
Puchner et al., 2021 [59]	Prospective Single group	COV+:<br>n=23 (7f, 16m);<br>57±10 yo; 27±4 kg/m²<br>Overweight/obesity (n=15), CVD (n=11), Endocrine disease (n=11), HT (n=9), DM2 (n=6), Pulmonary disease (n=5), CKD (n=3), Cancer (n=3), Immunodeficiency (n=3), Asthma (n=3), Chronic liver disease (n=2), Hypercholesteromia (n=2), COPD (n=1), No comorbidity (n=3)	**Moderate-to-High**<br>Hospitalization: 32±16 days<br>ICU: 19±14 days<br>Need for oxygen therapy (n=20)<br>Mechanical ventilation (n=19)	Immediately after hospital discharge 44±33 days after onset of infection (range: 20-70 days)	In hospital (rehabilitation clinic)<br><br>Physical activity, respiratory exercises, logopaedics and swallow evaluation, nutritional counseling, passive therapy sessions (*e.g.* massage)<br><br>Duration: 3-4 wk (24±5 days)<br>Total duration for physical activity program: 441±204 min	Modality: aerobic<br>Type: MICT, HIIT<br>Frequency: NR<br>Volume: 20min (MICT) or 26min (HIIT)<br>Intensity:<br>- MICT: 50%P_{max}<br>- HIIT: 8min 30%P_{max} (warm-up), 12sets x [30s 100%P_{max}/30s 0-20%Pmax], 6min 20%P_{max} (cooling-down)<br>[Evolution: RPE (Borg scale 6-20) was used to ease or progress intensity]<br>(P_{max} based on CPET)<br><br>Modality: resistance<br>Type: machines, bodyweight, elastic bands, dumbbells<br>Frequency: NR<br>Volume: NR<br>Intensity: NR	Functional capacity:<br>6MWT<br><br>Respiraroty function:<br>FVC, FEV1, FEV1/FVC, TLC, RV, DLCO, PI_{max}, PaO_2, $PaCO_2$, pH (blood samples, plethysmography, spirometry)<br><br>Quality of life, mental health, and general state:<br>Disability level (BI)
Rodriguez-Blanco et al., 2021 [60]	Prospective Controlled Trial (Pilot), two groups	COV+:<br>n=18 (9f, 9m);<br>39.4±11.7[µ] yo (f: 42.3±11.5 yo; m: 36.4±11.9 yo); NR<br>No comorbidity<br><br>CON (COV+ without RP):<br>n=18 (10f, 8m);<br>41.3±12.1 yo (f: 41.1±11.4 yo; m: 41.6±13.8 yo); NR<br>No comorbidity<br><br>Groups comparison: age (p<0.1)<br><br>Subjects with previously comorbidities (*e.g.* CKD, CVD, neurological disorders), or patients received a physical treatment in the previous 3 mo were excluded.	**Low**<br>No hospitalization (mild-to-moderate symptoms)	During the acute phase	Telerehabilitation (supervised and no-supervised)<br><br>Physical activity<br><br>Duration: 7 days	Modality: resistance<br>Type: 10 exercises based on non-specific toning exercises (free weight or light weights such as water bottles)<br>-<br>https://www.fisiosurid.com/ejercicios-proyecto-COVID/<br>Frequency: 1x/day, 7x/wk<br>Volume:<br>- 4rep if RPE=7-10 (Borg scale 0-10) on the 30-s STST (10min)<br>- 8rep if RPE=5-7 on the 30-s STST (20min)<br>- 12rep if RPE=1-5 on the 30-s STST (30min)<br>Intensity: NR<br><br>Supervised sessions 2x/wk by visioconference + text message daily for follow-up. No combination of the following program with other physical therapy or sports physical activity.	*Tests were performed pre- and post-RP, and 1 wk post-RP.*<br><br>Functional capacity:<br>6MWT (self-administrated by the patient at home using the app "StepsApp" which recorded the number of steps), 30-s STST, RPE post-30-s STST (Borg scale, probably 0-10)<br><br>Patients were instructed and telematically controlled by the study evaluator who provided patients the necessary assessment materials.
Saeki et al., 2021 [61]	Prospective Case series	COV+:<br>n=4 (m); 55.8±17.3 yo (range: 30-67); 26.5±1.7 kg/m²<br>HT (n=2), DM2	**High**<br>Hospitalization: 32±2 days (range: 29-34)<br>ICU: 19±2 days (range: 16-21)	During hospitalization (started in ICU)	In hospital and then telerehabilitation (no-supervised)<br><br>Physical activity (from	Before hospital discharge:<br>Modality: aerobic<br>Type: stepping, gait exercises, cycling (ergocycle)<br>Frequency: NR	*Tests were performed at ICU discharge, at hospital discharge, 1 mo and 6 mo after hospital discharge.*<br><br>Functional capacity:<br>6MWT, Muscle strength (MRC score 1-60), Gait speed

		(n=1), Bullous pemphigoid (n=1), No comobidity (n=2)	Mechanic ventilation: 16±3 days (range: 13-19)		ICU to the end of rehabilitation), positioning and postural drainage, passive exercises (from ICU to hospital discharge)<br><br>Duration: approximately 7 mo	Volume: NR<br>Intensity: NR<br><br>Modality: resistance<br>Type: muscle power training<br>Frequency: NR<br>Volume: NR<br>Intensity: NR<br><br><u>Total (for all modalities before hospital discharge):</u><br>Frequency: 1x/day<br>Volume: 40min<br><br><u>After hospital discharge (telerehabilitation):</u><br>Modality: aerobic<br>Type: walking<br>Frequency: NR<br>Volume: NR<br>Intensity: NR<br><br>Modality: resistance<br>Type: lower limbs (closed kinetic chain exercises), upper limbs (Thera-Band)<br>Frequency: NR<br>Volume: NR<br>Intensity: NR<br><br>Modality: balance<br>Type: NR<br>Frequency: NR<br>Volume: NR<br>Intensity: NR	<u>Quality of life, mental health, and general state:</u><br>Disability level (BI), SaO_2 pre- and post-6MWT
Spielmanns et al., 2021 a [62]	Retrospective Controlled trial, two groups	COV+ (tested positive for COVID-19 during RP and were temporary isolated):<br>n=27 (9f, 18m);<br>71.5±12.3 yo;<br>25.3±5.5 kg/m²<br>Stroke (n=19), HT (n=18), CKD (n=11), Dislipidemia (n=10), DM2 (n=9), Smoking history (n=8), COPD (n=6), Coronary artery disease (n=5), Peripheral artery disease (n=0)<br>CIRS: 17.2±6.6<br><br>COV- (needing RP before and during COVID-19 pandemia):<br>n=786 (399f, 387m);<br>71.5±13.1 yo;<br>25.9±5.8 kg/m²<br>Stroke (n=541), HT (n=512), Smoking history (n=347), CKD	**Low-to-Moderate**<br>Need for oxygen (COV+: n=10)	During the acute phase	In hospital (single-center inpatient rehabilitation center)<br><br>Physical activity, and if necessary, occupational, speech, and neuropsychological therapy<br>Specifically during isolation intervention (positive COVID-19 patients) if necessary: dysphagia beside assessments, respiratory exercises, psychological support, massages, nutrition and diabetes counseling, social services, etc.<br><br>Duration:<br>- COV+: 54±24 days<br>- CON: 32±18 days<br>Groups comparison: $p<0.001$	<u>Normal program (COV- and COV+ after negative COVID-19 swab):</u><br>Modality: aerobic<br>Type: NR<br>Frequency: NR<br>Volume: NR<br>Intensity: NR<br><br>Modality: resistance<br>Type: circuit training if possible<br>Frequency: NR<br>Volume: 3sets x 10-15rep<br>Intensity: 10-15RM<br><br>Modality: coordination<br>Type: circuit training if possible<br>Frequency: NR<br>Volume: 3sets x 10-15rep<br>Intensity: 10-15RM<br><br><u>Total (for all modalities for the normal program):</u><br>Frequency: 4-6x/wk for individual sessions + NR for group sessions<br>Volume: 30-45min<br><br><u>During isolation (only for COV+):</u><br>Modality: aerobic	<u>Quality of life, mental health, and general state:</u><br>Disability level (FIM)

		(n=301), Dislipidemia (n=259), DM2 (n=212), COPD (n=159), Coronary artery disease (n=159), Peripheral artery disease (n=89)<br>CIRS: 16.9±5.8				Type: cycling (ergocycle), stepping (stepper), and other ergometers<br>Volume: NR<br>Frequency: NR<br>Intensity: NR<br><br>Modality: resistance<br>Type: elastic bands (in patient's room)<br>Volume: NR<br>Frequency: NR<br>Intensity: NR<br><br><u>Total (for all modalities for the isolation program):</u><br>Frequency: 1-2x/day<br>Volume: 15-45min	
Spielmanns et al., 2021 b [63]	Prospective and retrospective (in function of group)<br>Controlled trial, two groups	COV+:<br>n=99 (42f, 57m);<br>67.7±10.2 yo;<br>28.2±6.1 kg/m²<br>HT (n=54), Smoking history (n=27), Overweight/obesity (n=25), Musculoskeletal disease (n=25), Dyslipidemia (n=20), Neurological disease (n=20), CKD (n=19), Coronary artery disease (n=18), Cancer (n=15), COPD (n=11), Cerebrovascular insufficiency (n=9), Atrial fibrillation (n=8), Obstructive sleep apnea (n=7), Chronic heart failure (n=6), Venous thromboembolism (n=5), Interstitial lung disease (n=5), Liver disease (n=5)<br>In average 3 comorbidities/patient<br>CIRS: 14.2±5.9<br><br>COV- (patients with lung disease in year 2019):<br>n=419 (213f, 206m);<br>69.3±11.3 yo;<br>24.5±6.1 kg/m²<br>CIRS: 14.5±5.6<br><br>Groups comparison:<br>BMI ($p<0.001$)	**Moderate-to-High**<br>Hospitalization (included ICU): 26±9 days<br>ICU (n=65): 11±13 days<br>Ventilation: 7±9 days<br><br>Symptoms/complications due to COVID-19: Sepsis (n=37), Delirium (n=36), Acute respiratory distress syndrome (n=31), Weakness due to ICU (n=24), Anemia (n=24), Electrolyte disturbance (n=18), Acute renal failure (n=18), Atrial fibrillation (n=13), Myocarditis (n=12), Hepatitis (n=12), Venous thromboembolism (n=11), Bacterial superinfection (n=11), DM (n=8), HT (n=6), Acute heart failure (n=4), COPD (n=1), Coronary artery disease (n=1), Pulmonal artery disease (n=1)	Immediately after hospital discharge, after being asymptomatic for 2 days and 10 days after onset of infection	In hospital (single-center inpatient rehabilitation center)<br><br>Physical activity, respiratory and relaxation exercises, education (1h twice a wk), and if necessary, smoking cessation program, psychosocial support or diabetes advices<br><br>Duration:<br>- Total groups: 3 wk (25-30 therapy sessions on 5-6 weekdays)<br>- COV+: median: 20 days [IQR: 18, 22]<br>- CON: median: 21 days [IQR: 18, 21]<br>Groups comparison: $p<0.05$	Modality: aerobic<br>Type: cycling (IT on ergocycle), walking (IT on treadmill) (Partially bedridden patients: started with in-bed cycling, walking with aids and then IT cycling)<br>Frequency: 5-6x/wk<br>Volume: 5-35min [Evolution: increase of duration in function of patient level]<br>Intensity:<br>- 6MWT<200m (n=58): cycling only, 30-60s at 55-70%HR_{max} / 60s low intensity (RPE=4-6, Borg scale 0-10)<br>- 6MWT>200m: 80% of 6MWT speed (RPE<6)<br>Interrupted exercise if SaO_2<88% or RPE>6.<br>(NR if HR_{max} is estimated or previously evaluated)<br><br>Modality: aerobic<br>Type: outdoor walking<br>Frequency: 2-3x/wk<br>Volume: 45min<br>Intensity: level 1 (slow pace with little incline of the terrain) to level 3 (faster pace with frequent inclines)<br><br>Modality: resistance<br>Type: large muscle groups<br>Frequency: 3-4x/wk<br>Volume: 30min, 3sets x 8-12rep per exercise (3-5 exercises in total)<br>Intensity: NR<br><br>Modality: 'gymnastics'<br>Type: mixture of exercises to improve endurance, strength, coordination range of motion, balance<br>Frequency: 5-6x/wk<br>Volume: 45min<br>Intensity: level 1 (sitting position with several breaks) to level 3 (standing position or walking with very few or no breaks)<br><br><u>Total (for all modalities):</u>	<u>Functional capacity:</u><br>6MWT<br><br><u>Quality of life, mental health and general status:</u><br>Disability level (FIM), Wellbeing (feeling thermometer)

						Frequency: max 4x/day for Monday to Friday, 1x/day for Saturday	
Stavrou et al., 2021 [64]	Prospective Single group	COV+:<br>n=20 (5f, 15m); 64.1±9.9 yo; 30.3±4.3 kg/m²<br>HT (n=13), DM (n=4), Smoking history (n=2), COPD (n=2), CVD (n=2)	**Moderate-to-High**<br>Hospitalization: 15±15 days<br>ICU (n=4)	2 mo after hospital discharge	Telerehabilitation (no-supervised)<br><br>Physical activity, stretching, respiratory exercises (yoga), nutrional personalized recommendations<br><br>Duration: 8 wk	Modality: aerobic<br>Type: walking (on flat and hard surface)<br>Frequency: 3x/wk<br>Volume: 50min<br>Intensity: initially 75%HR_{peak} (wk 1) and increase until 110%HR_{peak} (wk 8) [Evolution: +5% every wk] / initially leg fatigue=2 (Borg scale 0-10) and dyspnea=3 (Borg scale 0-10) (wk 1) [Evolution: increase until leg fatigue=6 and dyspnea=6 (wk 7)] (HR_{peak} evaluated during 6MWT)<br><br>Modality: resistance<br>Type: dumbell side lateral raises (a), dumbbell squats (b), chair lunges (c), seated leg raises (d), elbow flexion-extension on the chest with medecin ball (e)<br>Frequency: 3x/wk<br>Volume: 2sets x 12rep for (a) and (b) (wk 1-2) [Evolution: increase until 3sets x 16rep for all exercises (wk 8)] (20min)<br>Intensity: (a): 1.5kg, (e): 2kg, (b), (c), (d): body weight<br><br>Warm-up and recovery for each session: 3 exercises of stretching	<u>Functional capacity:</u><br>6MWT, HR, blood pressure pre-, post- and 1min post-6MWT, Lower extremity fatigue (Borg scale) pre- and post-6MWT, HR during 6MWT, 30-s STST, HR pre- and post-30-s STST, Handgrip<br><br><u>Respiratory function:</u><br>FVC, FEV1 and DLCO (spirometry, pneumotachograph), Dyspnea (Borg scale 0-10) pre- and post-6MWT, SaO_2 pre-, post- and 1min post-6MWT, SaO_2 during 6MWT, SaO_2 pre- and post-30-s STST, Chest circumference difference between maximal inhalation and exhalation<br><br><u>Quality of life, mental health and general status:</u><br>Sleep quality (PSQI)<br><br><u>Biological parameters:</u><br>ROS and Plasma antioxidant capacity (blood samples)<br><br><u>Other:</u><br>BMI, Neck circumference, Circumference differences between right and left Arm, Thigh, and Calf, FM, Visceral FM and Muscle mass (impedancemetry), Rest metoblic rate (method NR)
Steere et al., 2021 [65]	Prospective Three groups	Total groups (COV+):<br>n=102 (44f, 58m); 59.5±13.7 yo; NR<br>HT (n=56), Obesity (n=50), Smoking history (n=28), CVD (n=26), Chronic lung disease (n=16), CKD (n=15), Depression (n=14), Other mental health (n=12), Immunosuppression (n=9), Cancer (n=5), Dementia (n=2), Liver disease (n=1)<br><br>Participants were divided in 3 groups in function of the rehabilitation location, but we extracted the results only for all groups.	**Moderate-to-High**<br>Hospitalization: 15.2±10.8 days<br>ICU (n=38)<br>Mechanical ventilation (n=36): 14.9±6.4 days	Unclear	Telerehabiltation (supervised and no-supervised)<br><br>Physical activity, respiratory exercises, advices for mental health In addition, psychologist telehealth, occupational and speech therapy were coordinated based on identified patient needs.<br><br>Duration: 14.6±9.6 days	Modality: aerobic<br>Type: walking<br>Frequency: 5x/day (wk 1), 3x/day (wk 2), 2x/days (wk 3)<br>Volume: 5min (wk 1), 10min (wk 2), 15min (wk 3)<br>Intensity: NR<br><br>Modality: resistance<br>Type: circuit training detailed in a guide for the patients (sit to stand, standing walk, shoulder caption, standing heel raises, sidestepping, wall pushups) or detailed in videos on Internet (for phase 4)<br>Frequency: 1 cycle 4x/day everydays (phase 1); 2 cycles 2x/day, 6x/wk (phase 2); 3 cycles 2x/day, 6x/wk (phase 3)<br>Volume: 1 to 3 cycles; alternance of 30s work/30s rest (30-60min)<br>Intensity: NR, but HR, SaO_2 (always>88%) and RPE (Borg scale 0-10) were monitored.	<u>Functional capacity:</u><br>2-min step test, 30-s STST<br><br><u>Respiratory function:</u><br>SaO_2 pre- and post-functional tests (2-min step test and 30-s STST)
Sun et al., 2021 [66]	Prospective Single group	COV+:<br>n=31 (12f, 19m); 60.4±10.2 yo; NR<br>HT (n=12), DM (n=2), Hepatitis B (n=2), Cancer (n=2),	**Moderate-to-High**<br>Patients with respiratory rate≥30/min or SaO_2≤93% when they were breathing ambient air or PaO_2/FiO_2≤300mmHg<br><br>Abnormalities on chest (CT): Bilateral	During the acute phase	In hospital (by video teaching in the isolation ward)<br><br>Physical activity,	Modality: resistance<br>Type: upper limbs (upward lift, lateral lift, abduction, chest enlargement, grasping), lower limb (lifting, kicking, tiptoeing, stepping, bridge exercise, air pedal, ankle pump). All	*Tests were performed at pre-RP, after 2 wk and after 3 wk of RP.*<br><br><u>Functional capacity:</u><br>Exercise tolerance (capacity to elevate legs in bed, to stand and sit), Activities of daily living (capacity to wear clothes, to wear shoes, to go to the toilet by himself)

		CKD (n=2), Coronary heart disease (n=2), Tuberculosis (n=1), Smoking history (n=0), No comorbidity (n=9)<br><br>Previous history of rehabilitation: n=7	patchy shadowing (n=21), Ground-glass opacity (n=16), Pulmonary bullae (n=1)<br><br>COVID-19 symptoms at RP admission: Fever (n=23), Cough (n=22), Shortness breath (n=12), Sputum production (n=4), Diarrhea (n=2), Fatigue (n=1)		respiratory exercises, psychotherapy<br><br>Duration: 3 wk	exercises were performed in lying, sitting and standing positions<br>Frequency: 2x/day<br>Volume: for each movement, 5s x 2-3rep<br>Intensity: NR (but always SaO_2>93%, HR<120bpm - if palpitation, sweating, shortness of breath: activity stopped)<br><br>Patients who needed oxygen therapy could do it in bed or at bedside.	Respiratory function:<br>Dyspnea (mMRC Dyspnea Scale), SaO_2, Oxygen intake (probably from exogenous source), Need for oxygen therapy, Sputum, Cough and Shortness of breath (Questionnaires not further defined)<br><br>Quality of life, mental health, and general state:<br>Sleep quality (method NR), COVID-19 symptoms (method NR)<br><br>Biological parameters:<br>Lymphocytes, ALB, Total cholesterol, Neutrophils, CRP, Procalcitonin, WB, RBC, Hemoglobin, Triglycerids (methods NR)<br><br>Other:<br>Dietary habits (method NR)
Tozato et al., 2021 [67]	Prospective Case series	COV+:<br>n=4 (2f, 2m);<br>56.0±12.1 yo; NR<br>HT (n=3), Smoking history (n=1), HIV (n=1), Cancer (n=1), No comorbidity (n=1)	**Moderate-to-High**<br>Hospitalization (duration indicated for n=3): 29±28 days (calculated from raw data)<br>ICU (n=1): 9 days	Immediately after hospital discharge (n=3), 107 days after the symptoms start (n=1)<br><br>One subject had tetraparesis and started an adapted rehabilitation program during 45 days before to begin the RP.	In hospital<br><br>Physical activity in association with other therapies (NR).<br><br>Duration: 3 mo	Modality: aerobic<br>Type: walking (treadmill), cycling (ergocycle), arm ergometer, stepping<br>Frequency: 3x/wk<br>Volume: 30min<br>Intensity: 60-80%$HR_{reserve}$ (estimated with Karvonen formula), RPE=4-6 (Borg scale 0-10), $SaO_2\geq$90%<br><br>Modality: resistance<br>Type: all muscle groups<br>Frequency: 3x/wk<br>Volume: 3sets x 10rep for each exercise<br>Intensity: 60%1RM	Functional capacity:<br>6MWT, Handgrip, HR_{max} during 6MWT, RPE_{max} during 6MWT (Borg scale 0-10), Double product (HR x SBP) at rest, 1RM knee extension, 1RM shoulder abduction, 1RM elbow flexion<br><br>Respiratory function:<br>SaO_{2min} during 6MWT
Udina et al., 2021 [68]	Prospective Single group (but constitution of subgroups for specific analyses)	Total groups (COV+):<br>n=33 (19f, 14m);<br>66.2±12.8 yo; NR<br>Comorbidities/patient: 1.5±1.6<br>Pre-COVID-19 well-functioning adults (low disability level, great indenpence level, low frailty status, low comorbidities)<br><br>ICU+ (COV+):<br>n=20 (10f, 10m);<br>58.2±7.9 yo; NR<br>Comorbidities/patient: 0.5±0.8<br><br>ICU- (COV+):<br>n=13 (9f, 4m);<br>78.4±8.1 yo; NR<br>Comorbidities/patient: 2.8±1.8<br><br>Subsample who performed 6MWT (ICU+ and ICU-):<br>n=22 (14f, 8m);<br>61.9±12.1 yo; NR<br><br>Comparison groups between ICU+ and ICU-: age (p<0.001),	**Moderate-to-High**<br>ICU (n=20 for Total groups; n=18 for subsample who performed 6MWT): 10.3±9.9 days<br>Mechanical ventilation (n=20, only ICU+)<br><br>Symptoms/complications due to COVID-19 at admission to RP: Pneumonia (Total groups: n=30; ICU+: n=20; ICU-: n=10; subsample who performed 6MWT: n=21)<br><br>Polypharmacy (≥5 drugs) at admission to RP (Total groups: n=24; ICU+: n=13; ICU-: n=11)	Immediately after post-acute care	In hospital<br><br>Physical activity, respiratory exercises, manual therapy if necessary, anti-sedentary advices<br><br>Duration: 8.2±1.7 days	Modality: aerobic<br>Type: stepping, cycling (ergocyle), walking (continuous for all)<br>Frequency: NR<br>Volume: 5-15min<br>Intensity: RPE=3-5 (Borg scale 0-10)<br><br>Modality: resistance<br>Type: upper and lower limbs<br>Frequency: NR<br>Volume: 2-4 exercises, 1-2 sets x 8-10rep<br>Intensity: 30-80%1RM<br><br>Modality: balance<br>Type: static and dynamic (walking with obstacles, changing directions, unstable surfaces, unbalance, functional exercises)<br>Frequency: NR<br>Volume: 2 exercises<br>Intensity: N/A<br><br>+ recommendations to decrease daily sedentary behaviors<br><br>Total (for all modalities + breathing exercises):<br>Frequency: 1x/day, 7x/wk<br>Volume: 30min	Functional capacity:<br>6MWT (evaluated only in a subsample for logistical reasons), SPPB (score 0-12 from 3 tests: static body balance, ability to walk and stand up from a chair), Ambulation capacity (FAC 4-5), Standing balance on a single leg (Single leg stance test - maintaining single leg stance for 10s)<br><br>Quality of life, mental health, and general state:<br>Disability level (BI), Delirium (Confusion assessment method)

		comorbidities<br>(p<0.001)					
Zampogna et al., 2021 [69]	Prospective Single group	COV+:<br>n=140 (45f, 90m);<br>median: 71.0 [IQR: 61.5, 78.0] yo;<br>median: 25.2 [IQR: 23.2, 29.3] kg/m²<br>COPD (n=23),<br>Asthma (n=6)	**Moderate-to-High**<br>'Patients transferred from ICU, sub-ICU, pneumology units, or general wards'<br>Hospitalization: median: 47 [IQR: 33.5, 64] days<br>Respiratory status during hospitalization: Invasive ventilation (n=56), Non-invasive ventilation (n=70), Need for oxygen therapy (n=117)<br><br>Respiratory status at RP admission: Need for oxygen therapy (n=33)	NR	In hospital (inpatient)<br><br>Physical activity, drug therapy, and in function of patients' need, chest physiotherapy, lung expansion procedures, nutritional and psychological therapy<br><br>Duration: median: 24 [IQR: 19, 34] days (total sessions - median: 60)	Level A (SPPB<6):<br>Modality: aerobic<br>Type: free walking<br>Frequency: NR<br>Volume: NR<br>Intensity: NR<br><br>Modality: resistance<br>Type: peripheral limbs, shoulders, full arm circling<br>Frequency: NR<br>Volume: NR<br>Intensity: NR<br><br>Level B (SPPB≥6):<br>Modality: aerobic<br>Type: paced walking, cycling (ergocycle, for patients with higher physical autonomy)<br>Frequency: NR<br>Volume: NR<br>Intensity: for cycling, low intensity (<3 METs), RPE=4-5 (Borg scale 0-10)<br><br>Modality: resistance<br>Type: gymnastics, strengthening<br>Frequency: NR<br>Volume: NR<br>Intensity: NR<br><br>Modality: balance<br>Type: NR<br>Frequency: NR<br>Volume: NR<br>Intensity: NR<br><br>Total (for all modalities):<br>Type: without devices or used of gymnastic tools such as balls, canes, balance boards, or light weights bands<br>Frequency: 1-3x/day<br>Volume: 20-30min<br>Intensity: NR [Evolution: subjects were assessed daily to adjust the type, intensity, timing and modality of the intervention]	Functional capacity:<br>6MWT, SPPB (score 0-12 from 3 tests: static body balance, ability to walk and stand up from a chair)<br><br>Quality of life, mental health, and general state:<br>Disability level (BI)

10MWT: 10-minute walk test, 6MWT: 6-minute walk test, ALB: albumin, AMPAC: activity measure for post-acute care 6 clicks, ASM: appendicular skeletal muscle mass, BI: Barthel index, BMI: body mass index, BNP: brain natriuretic peptide, bpm: beats per minute, CIRS: cumulative illness rating scale, CKD: chronic kidney disease, CO_2: carbon dioxide, CON: controlled group (COVID-19 subjects who didn't performed rehabilitation program), COPD: chronic obstructive pulmonary disease, COV+: COVID-19 subjects who performed rehabilitation program, COV-: subjects without COVID-19 infection who performed rehabilitation program, CPET: cardiopulmonary exercise test, CRP: C-reactive protein, CT: computed tomography, CVD: cardiovascular disease, DLCO: diffusing capacity for carbon monoxide, DM: diabetus mellitus, DBP: diastolic blood pressure, EI: independent exercise group, EQ5D: EuroQol questionnaire - 5 dimensions, ESWT: endurance shuttle walking test, f: female subjects, FAC: Functional ambulatory category, FEV1: forced expiratory volume in 1 second, FFM: fat-free mass, Fi: fraction of inspired gas, FIM: functional independence measure, FM: fat mass, FVC: forced vital capacity, GAD-7: generalized anxiety disorder-7 questionnaire, HADS: hospital anxiety and depression scale, HR: heart rate, HIIT: high intensity interval training, HPT: home in-person physical therapy group, HT: hypertension, ICU: intensive care unit, IL: interleukine, Ig: immunoglobin, IQR: interquartile range, IT: interval training, KCO: carbon monoxide transfer coefficient, LDH: lactate dehydrogenase, m: male subjects, MICT: moderate interval continuous training, min: minute, mMRC: modified Medical Research Council, mo:month, MoCA: Montreal cognitive assessment, MRC: Medical Research Council, N/A: not applicable, NR: not reported, NRS: numerical rating scale, O_2: oxygen, Pa: partial pressure of a gas, PEF: peak expiratory flow, PE_{max}: maximal expiratory pressure, PHQ-9: patient health questionnaire 9, PI_{max}: maximal inspiratory pressure, P_{max}: maximal puissance, PSQI: Pittsburgh sleep quality index, QoL: quality of life, RBC: red blood cells, rep: repetitions, RM: repetition maxima, ROS: reactive oxygen species, RP: rehabilitation program, RPE: rating of perceived exertion, RV: residual

volume, s: second, SaO_2: oxygen saturation, SARS-CoV-2: severe acute respiratory syndrome coronavirus 2, SBP: systolic blood pressure, SF-36: short form 36 health survey questionnaire, SPPB: short physical performance battery, STST: sit-to-stand test, TLC: total lung capacity, TNF-α: Tumor Necrosis Factor-alpha, VAS: visual analog scale, VC: vital capacity, VPT: virtual physical therapy group, WR: work rate, yo: year old. µ: unclear. Severity of COVID-19: Low: no-hospitalized patients, Moderate: hospitalized patients but not in intensive care, High: hospitalized patients in intensive care.

Supplementary Table S3: Effects of the rehabilitation program on function capacity, respiratory function, and quality of life in post-COVID-19 patients.

Reference	Effects of the RP on functional capacity	Effects of the RP on respiratory function	Effects of the RP on quality of life, mental health, and general state	Effects of the RP on Biological and other parameters and adherence and tolerability of the RP
Al Chikhanie et al., 2021 [35]	**6MWT (m):**<br>- COV+: 139±144 / 343±140*<br>- COV-: 137±152 / 223±171*<br>Groups comparison: COV+ > COV- in post-RP<br>**Handgrip (kg):**<br>- COV+: 18.1±8.0 / 23.5±8.5*<br>- COV-: NR<br>**Quadriceps isometric (kg):**<br>- COV+: 14.2±10.6 / 25.5±11.7*<br>- COV-: NR<br>**Tinetti test (score):**<br>- COV+: 25.0±3.0 / 27.5±1.0<br>- COV-: NR<br>Correlation between 6MWT improvement (m/days) with the number of days post-ICU (p=0.01), and with the number of days in RP (p=0.09).	**SaO_{2min} during 6MWT and Dyspnea post-6MWT:**<br>- COV+: →<br>- COV-: →<br>**FEV1, FVC, PI_{max}, PE_{max}:**<br>- COV+: ↗<br>- COV-: NR<br>**Need for oxygen therapy (% of sample):**<br>- COV+: 86 / 16%#<br>- COV-: 76 / 62%#	*Results only for COV+*<br>**QoL, Post-traumatic stress:** →<br>**Anxiety and depression, Fatigue:** ↘	<u>Adherence and tolerability:</u><br>**Adherence:** 100%
Bernal-Utrera et al., 2021 [36]	NR, but stated that patients felt daily improvement.	NR, but stated that patients felt daily improvement.	NR, but stated that patients felt significant improvements on physical symptomatology (fatigue, weakness, myalgia) and psychological benefits (more confidence, safeguarding, well-being, motivation, distraction from confinement and disease, emotional support from physical therapist' presence)	<u>Adherence and tolerability:</u><br>No apparent dropout. Great acceptability that 'could be related to the fact that patients felt that they took part in their recovery'
Bertolucci et al., 2021 [37]	**Peripheral nervous system impairment evaluated by electromyography (% of sample)**: 18 / 18%#	**Need for tracheostomy (% of sample):** 28 / 4%#<br>**Need for oxygen therapy (% of sample):** 79 / 21%#<br>**PaO_2/FiO_2 ratio:** 361±123 / NR	**Mental confusion (% of sample):** 18 / 0%#<br>**Disability level:** ↘<br>**Ambulation capacity:** ↗	<u>Drugs:</u><br>**Corticosteroids intake (% of sample):** 49 / 10%#<br>**Antipsychotic intake (% of sample):** 28 / 13%#<br><br><u>Other:</u><br>**Dysphagia (% of sample):** 18 / 0%#<br>**Rectal colonization:** →<br><br><u>Adherence and tolerability:</u><br>**Adherence:** 100%
Betschart et al., 2021 [38]	**6MWT (m):** 442 / 554***<br>**Δ6MWT (m):** +88 (range: 80-170)<br>For n=9/12: clinically significant ↗ 6MWT (>30m)	N/A	**QoL:** ↗<br>**Perception of COVID-19 specific limitations in daily life (n in sample):** 10 / 4# (negligible or slight limitations)	<u>Adherence and tolerability:</u><br>Dropout rate of 25% and 2 patients who stopped because they perceived their performance level and QoL have reached the level it was before the infection
Büsching et al., 2021 [39]	**6MWT (m):**<br>- COV+: 336±169 (n=41) / 484±147**** (n=45)<br>- COV-: 320±136 (n=48) / 417±145**** (n=50)<br>Groups comparison: COV+ > COV- in post-RP<br>**Δ6MWT (m):**<br>- COV+: +133±93 (n=40)<br>- COV-: +102±73 (n=47)<br>Groups comparison: COV+ > COV- (trend), COV+ ICU+ = COV+ ICU-	N/A	**Disability level:**<br>- COV+: ↘ (n=21)<br>- COV-: ↘<br>Groups comparison:<br>- pre-RP: COV+ (n=25) < COV-<br>- post-RP: COV+ (n=36) = COV-<br>- Δ: COV+ = COV-, COV+ ICU- = COV+ ICU-<br>**Disability level:**<br>- COV+: ↘ (n=45)<br>- COV-: ↘	<u>Adherence and tolerability:</u> no information

			Groups comparison:<br>- pre-RP: COV+ (n=45) < COV-<br>- post-RP: COV+ (n=45) < COV-<br>- Δ: COV+ = COV-, COV+ ICU- = COV+ ICU-	
Curci et al., 2021 [40]	**6MWT (m):** 240±81 (n=6) / 303±112* (n=37)<br>**Proportion 6MWT<200m in patients able to perform (% of sample):** 50 / 23%[#]<br>**RPE (probably post-6MWT) (Borg scale):** 16.0±2.3 / 12.2±2.5****	**Dyspnea in activity of daily life:** ↘<br>**Need for respiratory supports:** ↘ (all discharged patients needed no respiratory support)<br>**FiO_2:** ↘<br>**PaO_2/FiO2:** ↗<br>**PaO_2:** →<br>**SaO_2:** ↗[£]<br>**CT scan:** 74% showed a reduced ground glass opacity, 26% showed unchanged extension, 8% fully recovered	**Disability level:** ↘<br>**Ambulation capacity:** ↗ (recovered in 100% patients)	<u>Biological parameters:</u><br>**Arterial lactate, CRP, D-dimer, LDH:** ↘<br><br><u>Adherence and tolerability:</u><br>No dropout (but deaths of 2 patients)
Daynes et al., 2021 [41]	**Incremental shuttle walking test (m):** 300±198 / 413±229**<br>**ESWT (s):** 292±260 / 837±406**	**Respiratory symptoms:** ↘	**Fatigue:** ↘ (↗ for n=4)<br>**Anxiety and depression:** →<br>**QoL, Cognition:** ↗	<u>Adherence and tolerability:</u><br>**Adherence:** 94% (initially 32 subjects)<br>11/12 sessions completed in average
Dun et al., 2021 [42]	**6MWT (m):**<br>- COV+: 366±59 / NR[#]<br>- CON: 538±68 / NR[#]<br>**Δ6MWT (m):**<br>- COV+: +254±67<br>- Low-volume (≤17 RP sessions, n=14): +204<br>- High-volume (>17 RP sessions, n=13): +263<br>- CON: +60±58<br>Groups comparison: CON < COV+, Low-volume < High-volume	N/A	N/A	<u>Biological parameters:</u><br>COV+ = CON for all parameters changes (# for pre *vs* post-RP measures)<br><br><u>Adherence and tolerability:</u><br>Number of completed sessions: median: 17 (range: 5-30)
Everaerts et al., 2021 [43]	*All resuls expressed in median [IQR]*<br>*Pre-RP / 6 wk of RP / 12 wk of RP (post-RP)*<br>**6MWT (m):** 453 [342-529] / 549 [478-620]***[µ] / 605 [497-655]**[µ]<br>**6MWT (%pred):** 63 [53-73] / 77 [73-85]***[µ] / 88 [76-91]**[µ]<br>**Δ6MWT (m):** N/A / +86 [53-175] / +149 [90-221]<br>**Handgrip (%pred):** 69 [61-81] / 90 [78-103]***[µ] / 104 [96-112]**[µ]<br>Groups comparison ICU+ *vs* ICU-: improvements appeared more pronounced in ICU+ for 6MWT and Handgrip.<br>*Pre-RP / Post-RP*<br>**CPET work load (%pred):** 65 [50-78] / 96 [83-114]**[µ]<br>**VO_{2peak} estimated from CPET (ml/min/kg):** 16 [13-20] / 20 [16-30]**[µ]<br>**VO_{2peak} estimated from CPET (%pred):** 66 [56-73] / 91 [82-108]**[µ]<br>**Quadriceps force (%pred):** 61 [50-70] / 74 [71-87]**[µ]	**FVC, FEV1, TLC, DLCO, PI_{max}, PE_{max} (all in %pred):** ↗	**Anxiety and depression, Cognition:** →[µ]<br>**Cognitive deficits:** ↘[µ]	<u>Adherence and tolerability:</u><br>**Dropouts at 6 wk:** n=1<br>**Dropouts at 12 wk:** n=6/22 (satisfaying results n=2, poor motivation n=1, interfering medical problems - myasthenia gravis, lumbar discus hernia, severe cognitive dysfunction n=3)
Ferraro et al., 2021 [44]	*Results were analysed (T tests) from raw data.*<br>**6MWT (m):** 173±73 / 313±26**<br>**Handgrip (kg):** 21±9 / 28±8*<br>**10MWT (s):** 11±5 / 8±3*<br>**SPPB (score 0-12):** 6±5 / 11±2*	N/A	*Results were analysed (T tests) from raw data.*<br>**Fatigue, Disability level:** ↘	<u>Adherence and tolerability:</u><br>No information but one patient excluded for missing data.
Gloeckl et al., 2021 [45]	*All resuls expressed in median [IQR]*<br>**6MWT (m):**<br>- M.COV+: 509 [426, 539] / 557 [463, 633]*** (Δ +48 [35, 113])<br>- S.COV+: 344 [244, 392] / 468 [374, 518]*** (Δ +124 [75, 145])<br>Groups comparison: M.COV+ > S.COV+ in pre-RP, M.COV+ < S.COV+ for Δ<br>**6MWT (%pred):**<br>- M.COV+: 70 [58, 80] / 81 [68, 91]*** (Δ +11 [5, 15])<br>- S.COV+: 53 [42, 58] / 71 [60, 83]*** (Δ +18 [11, 23])<br>Groups comparison: M.COV+ < S.COV+ for Δ<br><br>*Only S.COV+:*<br>**ESWT (m):** 430 [195, 758] / 980 [390, 1385]*** (Δ +550 [50, 725])<br>**ESWT (s):** 460 [217, 625] / 1200 [312, 1200]**** (Δ +740 [143, 789])<br>**HR pre-ESWT (bpm):** 86 [77, 98] / 85 [75, 95] (Δ -1 [-8, 4])<br>**HR post-ESWT (bpm):** 114 [99, 126] / 108 [97, 119] (Δ -6 [-12, 5])	*All resuls expressed in median [IQR]*<br>**Dyspnea post-6MWT (Borg scale):** → (for the two groups, NS for groups comparison)<br>**PaO_2, FVC (%pred), FEV1 (%pred):** ↗<br>Groups comparison: M.COV+ > S.COV+ for FEV1 at pre-RP, M.COV+ = S.COV+ for all Δ<br>**TLC (%pred), KCO (%pred), $PaCO_2$:** → (NS for groups comparison)<br>**DLCO (%pred):**<br>- M.COV+: →<br>- S.COV+: ↗<br>Groups comparison: M.COV+ = S.COV+ for Δ<br>**Need for oxygen therapy at rest:**<br>- M.COV+: →<br>- S.COV+: ↘<br>Groups comparison: M.COV+ = S.COV+ for Δ<br>**Need for oxygen therapy at exertion:**	**SF-36 physical component (score):** → (NS for groups comparison)<br>**SF-36 mental component (score):**<br>- M.COV+: →<br>- S.COV+: ↗<br>Groups comparison: M.COV+ < S.COV+ for Δ<br><br>*Only S.COV+:*<br>**Depression, Anxiety:** ↘<br>**Cognition:** ↗<br>**COVID-19 symptoms (% of sample):**<br>- Dyspnea: 85 / 73%[#]<br>- Fatigue: 73 / 58%[#]<br>- Cough: 54 / 35%[#]<br>- Cognitive impairment: 14 / 14%[#]	<u>Biological parameters:</u><br>**CRP, Leukocytes, Pro-BNP:** → (NS for groups comparison)<br>**D-dimer:**<br>- M.COV+: →<br>- S.COV+: ↗<br>Groups comparison: M.COV+ < S.COV+ for Δ<br><br><u>Adherence and tolerability:</u><br>Patients realized 94 to 100% of rehabilitation sessions.

	**Handgrip (kg):** 25 [18, 35] / 30 [20, 39]** (Δ +5 [3, 7])<br>**Maximal quadriceps strength (%pred):** 78 [49, 98] / 100 [68, 103]** (Δ +21 [6, 31])<br>**5-rep STST (s):** 13.3 [10.5, 15.5] / 10.3 [8.5, 13.2]*** (Δ -3.0 [-4.3, 0.3])	- M.COV+: →<br>- S.COV+: ↘<br>Groups comparison: M.COV+ = S.COV+ for Δ<br><br>*Only S.COV+:*<br>**Breathing frequency pre- and post-ESWT, Dyspnea during daily activities:** ↘<br>**SaO_2 pre-ESWT (%):** ↗<br>**SaO_2 post-ESWT (%):** ↗$^{£}$		
Gobbi et al., 2021 [46]	**Handgrip (kg):**<br>- Total S.COV+: 17.3±5.6 / 20.6± 7.2** (Δ +19.71%)<br>- m S.COV+: 19.9±6.1 / 23.9±6.9$^{£}$ (Δ +6.04%)<br>- f S.COV+: 13.8±2.9 / 16.1±2.3* (Δ +16.67%)<br>- Tot NS.COV+: 25.7±8.2 / 26.7±8.3 (Δ +3.89%)<br>- m NS.COV+: 31.2±10.8 / 33.5±7.2 (Δ +7.32%)<br>- f NS.COV+: 22.4±5.1 / 22.2±5.4 (Δ -0.89%)<br>**Time-up-to-go test (s):**<br>- S.COV+: 31.5±23.5 / 17.9±15.9* (Δ -26.83%)<br>- NS.COV+: 13.1±8.1 / 9.0±3.3* (Δ -31.29%)<br><br># for groups comparison	**SaO_2 in lying position:** → (for the two groups)<br>**SaO_2 in sitting position:** ↗ (but trend for NS.COV+)<br>**SaO_2 in standing position:**<br>- S.COV+: ↗<br>- NS.COV+: →<br>**SaO_2 post-Time-up-and-go test:**<br>- S.COV+: ↗$^{£}$<br>- NS.COV+: →<br>**Dyspnea in lying position:** ↘ (for the two groups)<br>**Dyspnea in sitting position:**<br>- S.COV+: →<br>- NS.COV+: ↘<br>**Dyspnea in standing position:** ↘ (for the two groups)<br>**Dyspnea post-Time-up-and-go test:** ↘ (but trend for S.COV+)<br><br># for groups comparison	N/A	<u>Biological parameters:</u><br>**ALB:** → (for the two groups)<br>Groups comparison: S.COV+ < NS.COV+ in pre-RP<br>**Ferritin:**<br>- S.COV+: ↘<br>- NS.COV+: →<br>Groups comparison: S.COV+ > NS.COV+ in pre-RP<br>**Vitamin D:**<br>- S.COV+: ↗<br>- NS.COV+: →<br>Groups comparison: S.COV+ < NS.COV+ in pre-RP<br><br><u>Other:</u><br>**BMI:** → (for the two groups)<br>**Phase angle:**<br>- S.COV+: →<br>- NS.COV+: ↗<br>**ASM:** ↗ (for the two groups)<br>- m: → (for the two groups)<br>- f: ↗ (for the two groups)<br># for groups comparison<br><br><u>Adherence and tolerability:</u> no information
Güler et al., 2021 [47]	N/A	**Need for oxygen therapy (% of sample):** 84 / 8%$^{\#}$	**Disability level:** ↘<br>**Functional status (% of sample):**<br>- Bedridden: 55 / 2%$^{\#}$<br>- Wheelchair: 22 / 5%$^{\#}$<br>- Mobilized with oxygen requirement: 20 / 8%$^{\#}$<br>- Mobilized with assistive device: 2 / 22%$^{\#}$<br>- Mobilized with no requirement: 0 / 62%$^{\#}$	<u>Adherence and tolerability:</u> no information
Hameed et al., 2021 [48]	**2-min step test (unit NR):**<br>- VPT: 44±23 / 73±26***<br>- HPT: 46±27 / 71±30*<br>- IE: 54±24 / 76±14*<br>- CON: 59±30 / 69±12<br>**30-s STST (unit NR):**<br>- VPT: 9±4 / 13±3***<br>- HPT: 8±3 / 12±1**<br>- IE: 10±5 / 13±3$^{£}$<br>- CON: 12±4 / 11±7<br><br># for groups comparison	**SaO_2 (probably at rest) (%):**<br>- Total groups: 96±3 (n=80) / 98±1% (n=33)$^{\#}$<br>- 'Follow-up population'^{µ}: ↗<br>Groups comparison: NS in pre- and post-RP	N/A	<u>Other:</u><br>**Participants working (% of sample):**<br>- VPT: 2 / 14%$^{\#}$<br>- HPT: 0 / 20%$^{\#}$<br>- IE: 6 / 0%$^{µ\#}$<br>- CON: 0 / 0%$^{\#}$<br><br><u>Adherence and tolerability:</u><br>50% of participants were lost for the follow-up visit.
Hayden et al., 2021 [49]	**6MWT (m):**<br>- Total groups (n=97): 419±127 (range: 13-663) / 530±100 (range: 175-740) (ES: large)<br>- Group A (n=52): 377±142 (range: 13-603) / 508±111 (range: 175-720) (ES: large)<br>- Group B (n=27): 459±79 (range: 270-590) / 555±80 (range: 406-675) (ES: large)<br>- Group C (n=18): 480±97 (range: 275-663) / 554±86 (range: 380-740) (ES: large)	**ΔDyspnea at rest (NRS):**<br>- Total groups (n=82): -0.7 [95%CI: -1.0, -0.3] (ES: small)<br>- Group A (n=43): -0.6 [95%CI: -1.1, -0.2] (ES: small)<br>- Group B (n=24): -0.6 [95%CI: -1.2, -0.0] (ES: small)<br>- Group C (n=15): -0.9 [95%CI: -2.2, -0.4] (ES: small)<br>Time (p<0.001), Group NS, Group x Time NS<br>**ΔDyspnea at extertion (NRS):**<br>- Total groups (n=82): -2.2 [95%CI: -2.7, -1.6] (ES: large)<br>- Group A (n=43): -2.4 [95%CI: -3.2, -1.6] (ES: large)	**Δ"How intense is your cough?" (NRS):**<br>- Total group (n=82): -0.9 [95%CI: -1.3, -0.4] (ES: small)<br>- Group A (n=43): -1.1 [95%CI: -1.7, -0.6] (ES: moderate)<br>- Group B (n=25): -0.3 [95%CI: -0.6, 0.2] (ES: very small)<br>- Group C (n=): -1.1 [95%CI: -3.0, 0.7] (ES: small)<br>Time (p<0.001), Group (p<0.05), Group x Time NS<br>**Δ"How big is the amount of phlegm that you expectorate?" (NRS):**<br>- Total groups (n=81): -0.5 [95%CI: -0.8, -0.2] (ES: small)	<u>Biological parameters:</u><br>**ΔLDH (U/I):**<br>- Total groups (n=56): -4.1 [95%CI: -11.9, 3.8] (ES: very small)<br>- Group A (n=30): -4.1 [95%CI: -16.9, 8.7] (ES: very small)<br>- Group B (n=17): -5.0 [95%CI: -17.2, 7.2] (ES: small)<br>- Group C (n=9): -2.1 [95%CI: -18.5, 14.2]

Time ($p<0.001$), Group ($p<0.01$), Group x Time ($p<0.05$)
Δ6MWT (m):
- Total groups: 111 [95%CI: 94, 127]
- Group A: 131 [95%CI: 107, 155]
- Group B: 96 [95%CI: 72, 120]
- Group C: 74 [95%IC: 33, 114]
Groups comparison: Groups A and B > C; Patients who were the more severely burdened at pre-RP achieved the higher improvement.
Correlation with Δ6MWT:
- Positive correlation with ΔPaO_2 ($p<0.01$), ΔCRP ($p<0.05$), ΔEQ5D ($p<0.05$), ΔEQ5D VAS ($p<0.05$)
- Negative correlation with ΔPI_{max} ($p<0.01$)

- Group B (n=24): -2.2 [95%CI: -3.4, -1.0] (ES: large)
- Group C (n=15): -1.5 [95%CI: -2.9, -0.1] (ES: moderate)
Time ($p<0.001$), Group NS, Group x Time NS
Δ"How burdening or debilitating is your dyspnea at rest?" (NRS):
- Total groups (n=81): -1.1 [95%CI: -1.4, -0.7] (ES: moderate)
- Group A (n=43): -1.0 [95%CI: -1.5, -0.5] (ES: moderate)
- Group B (n=23): -1.4 [95%CI: -2.2, -0.5] (ES: moderate)
- Group C (n=15): -0.7 [95%CI: -1.6, 0.1] (ES: small)
Time ($p<0.001$), Group ($p<0.05$), Time x Group NS
Δ"How burdening or debilitating is your dyspnea on extertion?" (NRS):
- Total groups (n=81): -2.1 [95%CI: -2.7, -1.5] (ES: moderate)
- Group A (n=43): -2.4 [95%CI: -3.2, -1.6] (ES: large)
- Group B (n=23): -2.4 [95%CI: -3.6, -1.2] (ES: large)
- Group C (n=15): -0.9 [95%CI: -2.1, 0.4] (ES: small)
Time ($p<0.001$), Group NS, Group x Time NS**ΔVC (%pred):**
- Total groups (n=103): 8.3 [95%CI: 4.9, 11.8] (ES: small)
- Group A (n=54): 12.5 [95%CI: 7.3, 17.7] (ES: moderate)
- Group B (n=32): 6.7 [95%CI: 0.5, 12.8] (ES: small)
- Group C (n=17): -1.18 [95%CI: -6.27, 2.64] (ES: small)
Time ($p<0.01$), Group ($p<0.001$), Group x Time ($p<0.05$)
ΔDyspnea (mMRC score):
- Total groups (n=90): -0.7 [95%CI: -1.0, -0.5] (ES: moderate)
- Group A (n=48): -0.9 [95%CI: -1.3, -0.6] (ES: moderate)
- Group B (n=26): -0.5 [95%CI: -1.0, -0.0] (ES: small)
- Group C (n=16): -0.6 [95%CI: -1.0, -0.1] (ES: moderate)
Time ($p<0.001$), Group NS, Group x Time NS
ΔTLC (%pred):
- Total groups (n=103): 4.9 [95%CI: 2.3, 7.6] (ES: small)
- Group A (n=54): 6.5 [95%CI: 2.7, 10.4] (ES: small)
- Group B (n=32): 5.8 [95%CI: 0.6, 10.9] (ES: small)
- Group C (n=17): -1.7 [95%CI: -6.9, 3.4] (ES: very small)
Time ($p<0.05$), Group ($p<0.001$), Group x Time ($p<0.05$)
ΔFEV1 (%pred):
- Total groups (n=103): 8.6 [95%CI: 5.2, 12.0] (ES: small)
- Group A (n=54): 12.16 [95%CI: 6.5, 17.8] (ES: moderate)
- Group B (n=32): 6.4 [95%CI: 1.4, 11.5] (ES: small)
- Group C (n=17): 1.5 [95%CI: -1.6, 4.5] (ES: small)
Time ($p<0.001$), Group ($p<0.001$), Group x Time ($p<0.05$)
ΔFEV1/VC (%):
- Total groups (n=103): -0.2 [95%CI: -2.4, 2.1] (ES: very small)
- Group A (n=54): -1.0 [95%CI: -5.0, 2.9] (ES: very small)
- Group B (n=32): -0.3 [95%CI: -2.6, 2.1] (ES: very small)
- Group C (n=17): 2.8 [95%CI: 0.06, 5.5] (ES: moderate)
Time NS, Group NS, Group x Time NS
ΔDLCO (%pred):
- Total groups (n=93): 6.2 [95%CI: 4.0, 8.4] (ES: moderate)
- Group A (n=48): 9.9 [95%CI: 6.7, 13.1] (ES: large)
- Group B (n=30): 3.8 [95%CI: 0.48, 7.1] (ES: small)
- Group C (n=15): -1.0 [95%CI: -5.9, 3.9] (ES: very small)
Time ($p<0.001$), Group ($p<0.001$), Group x Time ($p<0.001$)
ΔPI_{max} (%pred):
- Total groups (n=98): 7.7 [95%CI: 3.4, 12.0] (ES: small)
- Group A (n=52): 6.3 [95%CI: 1.1, 11.5] (ES: small)
- Group B (n=31): 12.0 [95%CI: 1.7, 22.3] (ES: small)
- Group C (n=15): 3.7 [95%CI: -3.2, 10.6] (ES: small)
Time ($p<0.01$), Group £, Group x Time NS
ΔPaO_2 (%pred):
- Total groups (n=100): 4.0 [95%CI: 2.6, 5.5] (ES: moderate)
- Group A (n=51): 5.6 [95%CI: 3.7, 7.5] (ES: moderate)
- Group B (n=31): 3.7 [95%CI: 0.9, 6.5] (ES: small)
- Group C (n=18): 0.06 [95%CI: 3.4, -3.5] (ES: very small)

- Group A (n=42): -0.4 [95%CI: -0.9, 0.0] (ES: small)
- Group B (n=24): -0.6 [95%CI: -0.1, -0.1] (ES: small)
- Group C (n=15): -0.7 [95%CI: -1.6, 0.4] (ES: small)
Time ($p<0.001$), Group NS, Group x Time NS
Δ"How strong is the pain that you experience?" (NRS):
- Total groups (n=83): -0.8 [95%CI: -1.3, -0.2] (ES: small)
- Group A (n=44): -0.8 [95%CI: -1.6, 0.0] (ES: small)
- Group B (n=24): -0.5 [95%CI: -1.5, 0.6] (ES: very small)
- Group C (n=15): -1.1 [95%CI: -2.6, 0.3] (ES: small)
Time ($p<0.05$), Group ($p<0.001$), Group x Time NS
ΔEQ5D 5 levels (Score 5-25):
- Total groups (n=82): -2.4 [95%CI: -3.0, -1.9] (ES: large)
- Group A (n=42): -2.9 [95%CI: -3.7, -2.2] (ES: large)
- Group B (n=25): -2.0 [95%CI: -2.9, -1.1] (ES: large)
- Group C (n=15): -1.7 [95%CI: -3.4, -0.0] (ES: moderate)
Time ($p<0.001$), Group NS, Group x Time NS
ΔEQ5D 5 levels VAS (Scale 0-100):
- Total groups (n=83): 18 [95%CI: 15, 21] (ES: large)
- Group A (n=43): 21 [95%CI: 16, 26] (ES: large)
- Group B (n=24): 15 [95%CI: 8, 21] (ES: large)
- Group C (n=16): 16 [95%CI: 8, 24] (ES: large)
Time ($p<0.001$), Group ($p<0.01$), Group x Time NS
ΔFatigue (score 0-10):
- Total groups (n=81): -1.7 [95%CI: -2.1, -1.3] (ES: large)
- Group A (n μ): -2.1 [95%CI: -2.6, -1.7] (ES: large)
- Group B (n=24): -1.4 [95%CI: -2.2, -0.7] (ES: large)
- Group C (n=15): -0.9 [95%CI: -2.1, 0.3] (ES: small)
Time ($p<0.001$), Group ($p<0.01$), Group x Time ($p<0.05$)
ΔDepression (score 0-27):
- Total groups (n=78): -4.3 [95%CI: -5.1, -3.5] (ES: large)
- Group A (n=42): -4.8 [95%CI: -6.0, -3.7] (ES: large)
- Group B (n=23): -3.8 [95%CI: -5.4, -2.3] (ES: large)
- Group C (n=13): -3.2 [95%CI: -5.4, -1.2] (ES: large)
Time ($p<0.001$), Group NS, Group x Time NS
ΔAnxiety (score 0-21):
- Total groups (n=77): -2.4 [95%CI: -3.2, -1.6] (ES: moderate)
- Group A (n=40): -2.9 [95%CI: -4.1, -1.8] (ES: large)
- Group B (n=22): -2.5 [95%CI: -1.1, -0.7] (ES: moderate)
- Group C: (n=15) -0.9 [95%CI: -3.3, 1.4] (ES: small)
Time ($p<0.001$), Group NS, Group x Time NS
Post-rehabilitation subjective health changed compared to pre-rehabilitation (-7 = Very much worse, 0 = Unchanged, +7 = Very much better):
- Total group: 4.3±2.1 (range: -6-7)
- Group A: 5.2±1.5 (range: 0-7)
- Group B: 4.0±1.4 (range: -1-7)
- Group C: 2.8±2.2 (range: -6-6)
Post-rehabilitation subjective health changed compared to pre-COVID-19 infection (-7 = Very much worse, 0 = Unchanged, +7 = Very much better):
- Total group: -1.6±3.6 (range: -7-7)
- Group A: -0.5±3.8 (range: -7-7)
- Group B: -1.9±2.5 (range: -5-5)
- Group C: -4.3±3.0 (range: -7-4)

(ES: very small)
Time NS, Group NS, Group x Time NS
ΔBNP (pg/ml):
- Total groups (n=61): -28.4 [95%CI: -100.1, -43.2] (ES: very small)
- Group A (n=32): -50.7 [95%CI: -190.2, -88.8] (ES: very small)
- Group B (n=18): -5.2 [95%CI: -20.4, 10.0] (ES: very small)
- Group C (n=11): -1.6 [95%CI: -22.7, 19.4] (ES: very small)
Time NS, Group NS, Group x Time £
ΔD-dimer (ng/ml):
- Total groups (n=69): -468 [95%CI: -735, -202] (ES: small)
- Group A (n=41): -765 [95%CI: -1195, -335] (ES: moderate)
- Group B (n=18): -20 [95%CI: -112, 71] (ES: very small)
- Group C (n=10): -57 [95%CI: -147, 32] (ES: small)
Time ($p<0.01$), Group ($p<0.05$), Group x Time ($p<0.01$)
ΔCRP (mg/ml):
- Total groups (n=71): -1.8 [95%CI: -2.8, 0.8] (ES: small)
- Group A (n=39): -2.6 [95%CI: -4.2, 0.9] (ES: moderate)
- Group B (n=21): -1.3 [95%CI: -2.8, 0.3] (ES: small)
- Group C (n=11): 0.0 [95%CI: -1.2, 1.2] (ES: very small)
Time ($p<0.05$), Group NS, Group x Time NS

<u>Adherence and tolerability (n NR):</u>
"How effective was the rehabilitation program for you?" (Scale 0-10):
- Total groups: 8.3±2.0 (range: 0-10)
- Group A: 8.9±1.4 (range: 4-10)
- Group B: 7.8±1.6 (range: 4-10)
- Group C: 7.1±2.9 (range: 0-10)
One patient transferred to psychatric hospital due to an acute psychosocial crisis not related to RP. One patient quited RP because of disc prolapse. One drop-out. All the other 105 patients participated at least 90% of RP sessions.

		Time (p<0.001), Group (p<0.05), Group x Time (p<0.01)<br>**Δ$PaCO_2$ (mmHg):**<br>- Total groups (n=100): -0.1 [95%CI: -0.7, 0.4] (ES: very small)<br>- Group A (n=51): 0.1 [95%CI: -0.7, 1.0] (ES: very small)<br>- Group B (n=31): -0.8 [95%CI: -1.7, 0.1] (ES: very small)<br>- Group C (n=18): 0.11 [95%CI: -1.4, 1.6] (ES: very small)<br>Time NS, Group NS, Group x Time NS<br>**ΔRV (%pred):**<br>- Total groups (n=103): -0.28 [95%CI: -5.8, 5.2] (ES: very small)<br>- Group A (n=54): -2.2 [95%CI: -10.5, 6.1] (ES: very small)<br>- Group B (n=32): 3.4 [95%CI: -5.6, 12.3] (ES: very small)<br>- Group C (n=17): -1.2 [95%CI: -15.8, 13.3] (ES: very small)<br>Time NS, Group (p<0.001), Group x Time NS<br>**ΔTotal specific airway resistance (%pred):**<br>- Total groups (n=103): -8.7 [95%CI: -6.3, 3.7] (ES: very small)<br>- Group A (n=54): -14.2 [95%CI: -37.0, 8.6] (ES: very small)<br>- Group B (n=32): 1.77 [95%CI: -9.4, 13.0] (ES: very small)<br>- Group C (n=17): -11.0 [95%CI: -21.2, -0.7] (ES: moderate)<br>Time NS, Group NS, Group x Time NS		
Hermann et al., 2020 [50]	**6MWT (m):**<br>- Total group: 231±154 / 361±135[$] (Δ +130±78)<br>- Ventilation+: 241±154 / 387±136*** (Δ +145±59)<br>- Ventilation-: 223±158 / 342±135*** (Δ +119±90)<br>Groups comparison: Ventilation+ = Ventilation- in pre- and post-RP and for Δ	**SaO_2 (probably at rest) (%):**<br>- Total groups: 92.7±2.7 / 96.0±2.3[#]<br>- Ventilation+: 92.4±2.2 / 96.0±2.8[#]<br>- Ventilation-: 92.9±3.1 / 96.1±2.1[#]<br>Groups comparison: Ventilation+ = Ventilation- in pre- and post-RP<br>**Need for oxygen therapy (n in sample):**<br>- Total groups: 21 / 7[#]<br>- Ventilation+: 11 / 4[#]<br>- Ventilation-: 10 / 3[#]<br>Groups comparison: Ventilation+ = Ventilation- in pre- and post-RP	**Patients' feeling (points):**<br>- Total group: Δ = +40[#]<br>- Ventilation +: ↗<br>- Ventilation -: ↗<br>Groups comparison: Ventilation+ = Ventilation- in pre-RP and for Δ, Ventilation + > Ventilation- (trend)	<u>Adherence and tolerability:</u><br>No patient died, no dropout mentioned. Stated that the program was performed safely. All patients return back home without professional nursing support.
Imamura et al., 2021 [51]	**Muscle strength (score 1-60):** 44±8 / 51±7**<br>**SPPB (n=16) (score 0-12):** 5.9±1.2 / 8.8±2.1	**PI_{max}, PE_{max} (n=10):** →	**Ambulatory capacity (% of sample):**<br>- Non-functional gait / unable to walk: 52 / 30%[#]<br>- Walking dependent on continuous manual contact to support body weight: 11 / 0%[#]<br>- Gait dependent on intermittent or continuous manual contact: 19 / 4%[#]<br>- Walking under supervision or verbal guidance: 19 / 19%[#]<br>- Independent walking on level terrain, with supervision in other environments: 0 / 26%[#]<br>- Independent walking anywhere, including stairs: 0 / 22%[#]<br>**Disability level:** ↘	<u>Other:</u><br>**FFM (n=18), Body weight, BMI:** →<br>**Dysphagia (% of sample):**<br>- No dysphagia: 48 / 70%[#]<br>- Mild dysphagia: 15 / 15%[#]<br>- Mild-Moderate dysphagia: 37 / 15%[#]<br>**Malnutrition (% of sample, n=24):**<br>- No malnutrition: 4 / 46%[#]<br>- Moderate: 54 / 29%[#]<br>- Severe: 42 / 25%[#]<br><br><u>Adherence and tolerability:</u><br>**Adherence:** 85% (n=23)
Li et al., 2021 [52]	*Results only for n=13/16 patients who were breathing spontaneously*<br>*Results analysed from raw data*<br>**Walking distance (not a 6MWT) (m):** 21±15 / 49±3***<br>**Muscle strength (score 1-60):** 57±5 / 58±3[£]<br>**Physical function (score):** 9.3±2.1 / 10.7±1.5**<br>**Mobility (score):** 46.3±11.2/ 73.2±26.3***	*Results analysed from raw data*<br>**PaO_2/FiO_2 ratio, PI_{max}, PI_{max} (%pred), PEF, PEF (%pred):** ↗<br>**Dyspnea at rest:** ↘	*Results analysed from raw data*<br>**Disability level:** ↘	<u>Adherence and tolerability:</u><br>No apparent dropout, no patient desaturated during or after either respiratory or mobility intervention, no adverse effects associated with physical therapist interventions.
Martin et al., 2021 [53]	**1-min STST (n rep):**<br>- COV+: 17.6±4.7 / NR[#] (|Δ| 10 (range: 5-19))<br>- CON: 13.6±7.6 / NR[#] (|Δ| 5 (range: -4-11))<br>Groups comparison: COV+ = CON in post-RP, COV+ > CON for |Δ| **HR pre-STST (bpm):**<br>- COV+: 91±17 / NR[#]<br>- CON: 86±15 / NR[#]<br>Groups comparison: COV+ = CON in post-RP<br>**HR post-STST (bpm):**	**Dyspnea pre-STST (Borg scale):**<br>- COV+: 0 / NR[#] (range: 0-3)<br>- CON: 2 / NR[#] (range: 0-5)<br>Groups comparison: COV+ = CON in post-RP<br>**Dyspnea post-STST (Borg scale):**<br>- COV+: 5 / NR[#] (range: 3-8) (|Δ| +2.5 (range: -2-7))<br>- CON: 5 / NR[#] (range: 1-10) (|Δ| +2 (range: 0-6))<br>Groups comparison: COV+ = CON in post-RP and for |Δ| **SaO_2 pre-STST (%):**<br>- COV+: 95.1±1.9 / NR[#]	N/A	<u>Adherence and tolerability:</u><br>One drop-out after first session for privacy reasons (not included in the final evaluation).

	- COV+: 111±19 / NR[#] (|Δ| 43±25)<br>- CON: 102±15 / NR[#] (|Δ| 23±12)<br>Groups comparison: COV+ = CON in post-RP, COV+ > CON for |Δ|	- CON: 94.8±1.9 / NR[#]<br>Groups comparison: COV+ = CON in post-RP<br>**SaO_2 post-STST (%):**<br>- COV+: 91.8±3.3 / NR[#]<br>- CON: 92.5±1.7 / NR[#]<br>Groups comparison: COV+ = CON in post-RP		
Mohamed et al., 2021 [54]	N/A	N/A	**QoL:** ↗ (for the two groups)<br>Groups comparison: COV+ > CON in post-RP	Biological parameters:<br>**Lymphocytes, leukocytes:** ↗ (for the two groups)<br>Groups comparison: COV+ > CON in post-RP<br>**IL-10:** ↗ (for the two groups)<br>Groups comparison: COV+ = CON<br>**IL-6:**<br>- COV+: ↗<br>- CON: →<br>Groups comparison: COV+ = CON<br>**IgA-S:**<br>- COV+: ↗<br>- CON: →<br>Groups comparison: COV+ > CON in post-RP<br>**TNF-α:**<br>- COV+: →<br>- CON: ↗<br>Groups comparison: COV+ = CON<br><br>Adherence and tolerability:<br>**Drop-out:** n=3 for COV+ and n=2 for CON
Ozyemisci Taskiran et al., 2021 [55]	*Results provided only for n=5 patients in the COV+ group and n=9 patients in the CON group and only in post-RP*<br>**Muscle strength (score 1-60):**<br>- COV+: median: 58 (range: 48-60)<br>- CON: median: 57 (range: 48-60)<br>Groups comparison: COV+ = CON<br>**Handgrip (kg):**<br>- COV+: median: 30 (range: 7-32)<br>- CON: median: 22 (range: 6-36)<br>Groups comparison: COV+ = CON<br>**Restricted range of motion (n in sample):**<br>- COV+: 1 (shoulder: n=1)<br>- CON: 2 (shoulder: n=1, finger flexion: n=1)<br>Groups comparison: COV+ = CON	N/A	*Results provided only for n=5 patients in the COV+ group and n=9 patients in the CON group and only in post-RP*<br>**Physical function:** COV+ > CON[£]<br>**Pain, Role limitations-physical, Role limitations-emotional, Mental health, Social functioning, Energy, General health:** COV+ = CON<br>**Tremor in the hands (n in sample):**<br>- COV+: 2<br>- CON: 8<br>Groups comparison: COV+ < CON[£]	Adherence and tolerability:<br>**Mortality:**<br>- COV+: n=8/18<br>- CON: n=5/17[µ]
Pancera et al., 2021 [56]	*Results analysed from raw data for n=6*<br>*T0 / T1 / T2*<br>**Muscle strength (score 0-60):** 51±2 / 55±3** / 59±2***<br>**SPPB (score 0-12):** 1±3 / 9±5**** / 12±0****	*Results analysed from raw data for n=6*<br>**PI_{max}:** →<br>**PE_{max}:** ↗[£]	*Results analysed from raw data for n=6*<br>**QoL:** ↗<br>**Disability level:** ↘	*Results analysed from raw data for n=6*<br>Other:<br>**BMI, Quadriceps girth left and right:** ↗<br><br>Adherence and tolerability:<br>**Drop-out:** one patient discharged early because of an acute gallbladder problem unrelated to the RP (not included in results).
Patel et al., 2021 [57]	**ΔDistance ambulated (m):** +224 [95%CI: 172, 276]*** (ES: large)	**Could breathe on room air (% of sample):** 68 / 81%[#]<br>**Need for oxygen on exertion (% of sample):** 23 / 14%[#]	**AMPAC for basic mobility and daily activities:** ↗<br>Groups comparison: intubated patients > non-intubated patients for Δ, patients receiving psychotherapy > patients without psychotherapy for ΔAMPAC for daily activities and trend for ΔAMPAC for basic mobility<br>**Ambulatory status (% of sample):** 66 / 92%[#]	Other:<br>80% of patients discharged at home, 9% were discharged to a skilled nursing facility or subacute rehabilitation facility, 6% required medical transfer off the unit to an acute medical floor, 5% were transferred to another unit/hospital for further rehabilitation.<br><br>Adherence and tolerability: no information
Piquet et al., 2021 [58]	Groups comparison: ICU+ = ICU-, LONG+ (>14 days in acute care, n=50) = LONG- (<14 days in acute care, n=50) in all pre-RP measures	**Respiratory rate pre-STST, SaO_2 pre- and post-STST:** →	**Disability level (n=89):** ↘<br>Groups comparison: ICU+ = ICU-, LONG+ = LONG- in pre-RP	Correlations:<br>- Positive correlation between disability level and STST frequency (n=51) at admission

	**10-rep STST (s) (n=51):** 30.7±3.5 / 28.7±9.3**<br>**STST Frequency x100 (Hz) (n=51):** 26.9±15.1 / 36.9±15.1*** (Δ +37%)<br>**HR pre-STST (bpm) (n=50):** 88±16 / 90±17<br>**HR post-STST (bpm) (n=50):** 103±21 / 103±21<br>**RPE pre-STST (Borg scale) (n=37):** 0.7±1.4 / 0.2±0.7<br>**RPE post-STST (Borg scale) (n=37):** 3.0±2.3 / 2.1±1.5* (Δ -30%)<br>**Handgrip left (kg):** 17.0±9.8 / 20.1±9.2***<br>**Handgrip right (kg):** 19.2±9.9 / 21.9±9.6*** (Δ +15%)<br>**Mean handgrip (left+right) (kg):** 18.1±9.8 / 20.9±9.5***<br>Groups comparison: ICU+ > ICU- for Δ<br>At discharge, mean grip strength remained 10% below normal values in that age range.	**Respiratory rate post-STST:** ↘<br>**Need for oxygen therapy (% of sample):** 58 / 3%[#]		(p<0.001[µ]) and at discharge (p<0.001[µ])<br>- Negative correlation between disability level and handgrip (n=48) at admission (p<0.01[µ]) and at discharge (p<0.01[µ])<br>- Negative correlation between handgrip and days spent in ICU (n=16) at admission (trend[µ]) and at discharge (p<0.05[µ])<br>- Negative correlation between handgrip and RPE post-STST at discharge (p<0.05)<br><br><u>Adherence and tolerability:</u><br>Death of 2 subjects. 79% of discharges home or to a relative's home, 15% of transfers to a COVID-free unit for further inpatient rehabilitation.
Puchner et al., 2021 [59]	**6MWT (m):** 323±196 / 499±103*** (Δ +176±137)	**FVC, FEV1, TLC, DLCO, PI_{max}:** ↗<br>**FEV1/FVC:** ↘<br>**RV:** ↗[£]<br>**pH:** ↘[£]<br>**PaO_2, $PaCO_2$:** →	**Disability level:** ↘	<u>Adherence and tolerability:</u><br>No information but 7 patients excluded due to missing data.
Rodriguez-Blanco et al., 2021 [60]	**6MWT (m):**<br>- COV+: 440±164 / 520±135* (Δ +80±126)<br>- CON: 380±129 / 380±136 (Δ 0±26)<br>Groups comparison: COV+ = CON in pre- and post-RP, COV+ > CON for Δ<br>**30-s STST (n rep):**<br>- COV+: 12.3±4.8 / 13.8±5.7* (Δ +1.5±2.2)<br>- CON: 10.5±2.3 / 9.9±2.0* (Δ -0.6±0.9)<br>Groups comparison: COV+ > CON in pre- and post-RP and for Δ<br>**RPE post-30-s STST (Borg scale):**<br>- COV+: 4.8±1.7 / 2.6±0.9*** (Δ -2.2±1.3)<br>- CON: 4.8±1.8 / 4.8±1.5 (Δ +0.1±1.3)<br>Groups comparison: COV+ = CON in pre-RP, COV+ > CON in post-RP and trend for Δ<br><br>No difference between females and males subjects for the two groups (details of data are available in article).	N/A	N/A	<u>Adherence and tolerability:</u><br>**Adherence:** 90%<br>Program found effective, safe, and feasible
Saeki et al., 2021 [61]	*Results analysed from raw data for parameters assessed in all subjects (n=4)*<br>**6MWT (m) from 1 mo to 6 mo:** 491±44 / 542±31*<br>**Muscle strength (score 1-60) from ICU discharge to 6 mo:** 52±3 / 57±3* / 60±1*** / 60±0***<br>**Gait speed (m/s) from hospital discharge to 6 mo:** 1.0±0.2 / 1.4±0.1 / 2.2±0.8*<br>**Handgrip right (kg) from 1 mo to 6 mo:** 23.8±10.6 / 33.9±5.6<br>**Handgrip left (kg) from 1 mo to 6 mo:** 24.9±4.0 / 31.6±6.8*	**SaO_2 pre- and post-6MWT from 1 mo to 6 mo:** →	**Disability level from ICU discharge to 6 mo:** ↘ (all patients achieved 100 (BI score) from 1 mo after hospital discharge)	<u>Adherence and tolerability:</u> no information
Spielmanns et al., 2021 a [62]	N/A	N/A	Groups comparison for **Disability level:** COV+ < COV- in pre-RP, COV+ = COV- in post-RP, COV+ < COV- for Δ # for pre *vs* post-RP	<u>Adherence and tolerability:</u><br>2 patients were transfered to acute hospital because of respiratory failure (one of them died).
Spielmanns et al., 2021 b [63]	**6MWT (m):**<br>- COV+: 176±141 / 357±132**** (Δ +180±101)<br>- COV-: 210±128 / 312±126**** (Δ +102±89)<br>Groups comparison: COV+ < COV- in pre-RP, COV+ > COV- in post-RP and for Δ	N/A	**Disability level:** ↘ (for the two groups)<br>Groups comparison: COV+ = COV- in pre-RP, COV+ < COV- in post-RP, COV+ > COV- for Δ<br>**Wellbeing:** ↗ (for the two groups)<br>Groups comparison: COV+ = COV- in pre-RP, COV+ > COV- in post-RP and for Δ	<u>Adherence and tolerability:</u> no information
Stavrou et al., 2021 [64]	**6MWT (m):** 434±102 / 519±95***<br>**6MWT (%pred):** 84±17 / 99±11***<br>**VO_{2peak} estimated from 6MWT (ml/min/kg):** 14.9±2.4 / 16.9±2.2***<br>**Metabolic equivalent estimated from 6MWT (METs):** 4.3±0.7 / 4.8±0.6***<br>**HR pre-6MWT (bpm):** 77±16 / 78±12<br>**HR post-6MWT (bpm):** 111±19 / 107±17[µ]	**Dyspnea pre-6MWT:** →<br>**Dyspnea post-6MWT:** ↘<br>**FVC, FEV1, DLCO (%pred):** →<br>**Chest circumference difference between maximal inhalation and exhalation:** ↗<br>**SaO_2 pre- and post-6MWT:** →<br>**SaO_2 1min post-6MWT:** ↘[µ]	**Sleep quality:** ↗	<u>Biological parameters:</u><br>**Plasma oxydant capacity:** ↗<br>**ROS:** →<br><br><u>Other:</u><br>**BMI, FM, Visceral fat, Neck circumference:** ↘

	**HR 1min post-6MWT (bpm):** 90±16 / 88±16<br>**HR 4e and 5e min of 6MWT:** pre-RP > post-RP$^{\mu}$<br>**HR (%pred$^{\mu}$):** 71±11 / 67±11*<br>**SBP pre-6MWT (mmHg):** 139±16 / 129±9**<br>**SBP post-6MWT (mmHg):** 160±14 / 152±12*<br>**SBP 1min post-6MWT (mmHg):** 142±12 / 138±8<br>**DBP pre-6MWT (mmHg):** 84±16 / 84±9<br>**DBP post-6MWT (mmHg):** 88±13 / 88±12<br>**DBP 1min post-6MWT (mmHg):** 83±12 / 83±9<br>**Lower extremity fatigue pre-6MWT (Borg scale):** 0.3±0.8 / 0.1±0.6<br>**Lower extremity fatigue post-6MWT (Borg scale):** 1.0±0.8 / 0.8±0.6<br>**30-s STST (n rep):** 11.4±3.2 / 14.1±2.7***<br>**HR difference pre/post 30-s STST (bpm):** 24±11 / 24±15<br>**Handgrip (kg):** 31.9±10.2 / 33.2±9.8	**SaO_2 4e min of 6MWT:** pre-RP < post-RP<br>**SaO_2 difference pre/post 30-s STST:** 1.7±1.3 / 1.5±2.3*		**Muscle mass:** ↗<br>**Body surface area, Rest metabolic rate, Circumference differences between right and left Arm, Thigh, and Calf:** →<br><br><u>Adherence and tolerability:</u><br>**Drop-out:** n=6 (initially 26 subjects)
Steere et al., 2021 [65]	NR	NR	N/A	<u>Adherence and tolerability:</u> no information
Sun et al., 2021 [66]	*Baseline (n=31) / 2 wk (n=29) / 3 wk (n=31)*<br>**Elevate legs in bed (n in sample):**<br>- Can: n=27 / n=28 / n=31<br>- Can't: n=4 / n=1 / n=0<br>Baseline *vs* 2 wk NS$^{\mu}$, Baseline *vs* 3 wk*$^{\mu}$<br>**Stand and sit by yourself (n in sample):**<br>- Can: n=27 / n=28 / n=31<br>- Can't: n=4 / n=1 / n=0<br>Baseline *vs* 2 wk NS$^{\mu}$, Baseline *vs* 3 wk*$^{\mu}$<br>**Wear clothes by yourself (n in sample):**<br>- Can: n=19 / n=28 / n=31<br>- Can't (short of breath slightly): n=7 / n=0 / n=0<br>- Can't (short of breath significantly): n=5 / n=1 / n=0<br>Baseline *vs* 2 wk**$^{\mu}$, Baseline *vs* 3 wk***$^{\mu}$<br>**Wear shoes by yourself (n in sample):**<br>- Can: n=21 / n=28 / n=31<br>- Can't: n=10 / n=1 / n=0<br>Baseline *vs* 2 wk***$^{\mu}$, Baseline *vs* 3 wk**$^{\mu}$<br>**Go to the toilet by yourself (n in sample):**<br>- Can: n=31 / n=29 / n=31 $^{\#}$<br>- Can't: n=0 / n=0 / n=0 $^{\#}$<br>*Details on statistical analysis between 2 wk and 3 wk are available in the article.*	**Dyspnea, Oxygen intake:** ↘<br>**SaO_2:** ↗<br>**Need for oxygen therapy, Whitish sputum:** ↘<br><br>*Baseline (n=31) / 2 wk (n=29) / 3 wk (n=31)*<br>**Productive cough (n in sample):**<br>- Easy: 6 / n=3 / NR<br>- Difficult: 3 / 2 / NR<br>- Can't cough phlegm: 8 / 1 / NR<br>Baseline *vs* 2 wk**$^{\mu}$<br>**When feel shortness breath (n in sample):**<br>- Prostration: 1 / 0 / 0<br>- Stroll: 12 / 3 / 0<br>- Scoot: 17 / 26 / 31<br>- No shortness breath: 1 / 0 / 0<br>Baseline *vs* 2 wk**$^{\mu}$, Baseline *vs* 3 wk***$^{\mu}$<br>*Details on statistical analysis between 2 wk and 3 wk are available in the article.*	*Baseline (n=31) / 2 wk (n=29) / 3 wk (n=31)*<br>**Sleep quality (n in sample):**<br>- Good: 5 / 6 / 24<br>- Wake up 1-2 times a night: 23 / 23 / 7<br>- Can't sleep: 3 / 0 / 0<br>Baseline *vs* 2 wk NS$^{\mu}$, Baseline *vs* 3 wk***$^{\mu}$<br>**Effect of cough on life (n in sample):**<br>- No effect: 15 / 7 / 1<br>- Marginal effect: 8 / 22 / 30<br>- Serious effect: 8 / 0 / 0<br>Baseline *vs* 2 wk NS$^{\mu}$, Baseline *vs* 3 wk NS$^{\mu}$<br>**When feel fatigue (n in sample):**<br>- Walk: 25 / 28 / 31<br>- Go to the toilet: 6 / 1 / 0<br>Baseline *vs* 2 wk$^{£\ \mu}$, Baseline *vs* 3 wk NS$^{\mu}$<br>**Whether palpitate (n in sample):**<br>- Never: 10 / 22 / 29<br>- Seldom: 20 / 7 / 2<br>- Usually: 1 / 0 / 0<br>Baseline *vs* 2 wk**$^{\mu}$, Baseline *vs* 3 wk***$^{\mu}$<br>*Details on statistical analysis between 2 wk and 3 wk are available in the article.*<br><br>*Baseline vs wk 2 and wk 3*<br>**Fatigue:** ↗<br>**Shortness breath, Cough, Sputum production:** ↘	<u>Biological parameters</u>:<br>*Baseline vs 2 wk*<br>**Lymphocytes, ALB, Total cholesterol:** ↗<br>**Neutrophils, CRP, Procalcitonin:** ↘<br>**WBC, RBC, Hemoglobin, Triglycerids:** →<br>*Baseline vs 3 wk*<br>**RBC, Hemoglobin, Lymphocytes, ALB, Total cholesterol:** ↗<br>**Neutrophils, CRP:** ↘<br>**Procalcitonin:** ↘$^{£}$<br>**WBC, Triglycerids:** →<br>*Details on statistical analysis between 2 wk and 3 wk are available in the article.*<br><br><u>Others:</u><br>*Baseline (n=31) / 2 wk (n=29) / 3 wk (n=31)*<br>**Dietary habits (n in sample):**<br>- As usual: 28 / 28 / 1$^{£}$<br>- Less than usual: 3 / 1 / 0$^{£}$<br><br><u>Adherence and tolerability:</u><br>In the first 3 days of rehabilitation: n=21 with side effects of muscle soreness and fatigue, but side effects disappeared within 1 wk.
Tozato et al., 2021 [67]	**6MWT (m):** 396±82 / 604±78*<br>**Handgrip right (kg):** 20.4±8.8 / 26.9±2.8<br>**Handgrip left (kg):** 18.6±6.9 / 25.2±4.2$^{£}$<br>**HR_{max} during 6MWT (bpm):** 131±16 / 148±14<br>**RPE_{max} during 6MWT (Borg scale):** 4.8±2.2 / 3.0±2.2<br>**Double product at rest (HR x SBP):** 12.1±1.3 / 9.9±2.0*<br>**1RM knee extension right and left (kg):** 7.3±5.3 / 15.5±7.1*<br>**1RM shoulder abduction right (kg):** 1.7±0.9 / 2.6±0.5*<br>**1RM shoulder abduction left (kg):** 1.6±0.9 / 2.4±0.5<br>**1RM elbow flexion right (kg):** 2.2±0.7 / 3.6±1.1$^{£}$<br>**1RM elbow flexion left (kg):** 2.1±0.5 / 3.4±0.8$^{£}$	**SaO_{2min} during 6MWT:** →	N/A	<u>Adherence and tolerability:</u> no information
Udina et al., 2021 [68]	**6MWT (m) (n=22):** 159±154 / 346±112***<br>**SPPB (score 0-12):**<br>- Total groups: 5.4±2.7 / NR$^{\$}$ (Δ +3.7±2.1)<br>- ICU+: 5.5±2.8 / NR$^{\$}$ (Δ +4.4±2.1)<br>- ICU-: 5.3±2.6 / NR$^{\$}$ (Δ +2.5±1.7)<br>Groups comparison: ICU- < ICU+ for Δ<br>**SPPB Balance (score 0-4):**<br>- Total groups: 2.8±1.3 / NR$^{\$}$ (Δ +0.8±1.1)	N/A	**Disability level:** ↘ (for the two groups, NS for groups comparison for Δ)<br>**Delirium:** although none of the patient had delirium at admission, post-COVID-19 cognitive function was mildly impaired in the whole cohort and within both groups	<u>Adherence and tolerability:</u><br>No death during the intervention and all were discharge home

	- ICU+: 2.7±1.3 / NR[$] (Δ +1.1±1.2)<br>- ICU-: 3.1±1.2 / NR (Δ +0.4±0.7)<br>Groups comparison: ICU- < ICU+ (trend) for Δ<br>**SPPB Gait speed (m/s):**<br>Total groups: 0.5±0.2 / NR[$] (Δ +0.3±0.2)<br>- ICU+: 0.5±0.3 / NR[$] (Δ +0.4±0.2)<br>- ICU-: 0.5±0.2 / NR[$] (Δ +0.2±0.1)<br>Groups comparison: ICU- < ICU+ for Δ<br>**SPPB Chair stand (s):**<br>- Total groups: 35.4±21.4 / NR[$] (Δ -14.1±16.9)<br>- ICU+: 33.7±21.1 / NR[$] (Δ -15.3±16.9)<br>- ICU-: 38.1±22.3 / NR[$] (Δ -12.2±17.6)<br>Groups comparison: ICU- = ICU+ for Δ<br>**Standing balance on a single leg (n in sample maintaining 10s):**<br>- Total groups: n=3 / n=13[$]<br>- ICU+: n=1 / n=10[$]<br>- ICU-: n=2 / n=1<br>Groups comparison: NR<br>**Unassisted gait (n in sample):**<br>- Total groups: n=19 / n=33[$]<br>- ICU+: n=13 / n=20[$]<br>- ICU-: n=6 / n=13[$]<br>Groups comparison: NR			
Zampogna et al., 2021 [69]	**6MWT (m) (n=42):** 229±103 / 328±98***[μ] (for n=81 at discharge: 298±117)<br>**6MWT (%pred) (n=42):** 48±19 / 68±15%***[μ]<br>**SPPB (score 0-12):** 3.2±3.7 / 6.9±3.8[$]	N/A	**Disability level:** ↘	<u>Adherence and tolerability:</u><br>31% of patients in level A, 62% shifted from leval A to level B, 7% in level B (70% of adherence in this level)

%pred: percentage of predicted value, 10MWT: 10-minute walk test, 6MWT: 6-minute walk test, 95%CI: 95% confidence interval, ALB: albumin, AMPAC: activity measure for post-acute care 6 clicks, ASM: appendicular skeletal muscle mass, BI: Barthel index, BMI: body mass index, BNP: brain natriuretic peptide, bpm: beats per minute, CO_2: carbon dioxide, CON: controlled group (COVID-19 subjects who didn't performed rehabilitation program), COV+: COVID-19 subjects who performed rehabilitation program, COV-: subjects without COVID-19 infection who performed rehabilitation program, CPET: cardiopulmonary exercise test, CRP: C-reactive protein, DLCO: diffusing capacity for carbon monoxide, DBP: diastolic blood pressure, EI: independent exercise group, EQ5D: EuroQol questionnaire - 5 dimensions, ES: effect size, ESWT: endurance shuttle walking test, f: female subjects, FAC: Functional ambulatory category, FEV1: forced expiratory volume in 1 second, FFM: fat-free mass, Fi: fraction of inspired gas, FIM: functional independence measure, FM: fat mass, FVC: forced vital capacity, HPT: home in-person physical therapy group, HR: heart rate, ICU: intensive care unit, IL: interleukine, Ig: immunoglobin, IQR: interquartile range, KCO: carbon monoxide transfer coefficient, LDH: lactate dehydrogenase, m: male subjects, min: minute, mMRC: modified Medical Research Council, mo: month, MRC: Medical Research Council, N/A: not applicable, NR: not reported, NRS: numerical rating scale, O_2: oxygen, Pa: partial pressure of a gas, PEF: peak expiratory flow, PE_{max}: maximal expiratory pressure, PI_{max}: maximal inspiratory pressure, QoL: quality of life, rep: repetition, RBC: red blood cells, RM: repetition maxima, ROS: reactive oxygen species, RP: rehabilitation program, RPE: rating of perceived exertion, RV: residual volume, s: second, SaO_2: oxygen saturation, SBP: systolic blood pressure, SF-36: short form 36 health survey questionnaire, SPPB: short physical performance battery, STST: sit-to-stand test, TLC: total lung capacity, TNF-α: Tumor Necrosis Factor-alpha, VAS: visual analog scale, VC: vital capacity, VPT: virtual physical therapy group, WBC: white blood cell, wk: week. Δ: change between pre-rehabilitation program and post-rehabilitation program, [μ]: unclear, NS: no significant difference, [£]p<0.1, * p<0.05, **p<0.01, ***p<0.001, ****p<0.0001, [$]significant but no p-value *versus* T0 (pre-RP measure), [#] no apparent statistics, >: significantly higher, < significantly lower, =: no statistical difference between groups. Effect size (d de Cohen's): <0.2 = very small, <0.5 = small, <0.8 = moderate, ≥0.8 = large.
